# Intravenous versus oral antibiotics for eradication of *Pseudomonas aeruginosa* in cystic fibrosis (TORPEDO-CF): a randomised controlled trial

**DOI:** 10.1016/S2213-2600(20)30331-3

**Published:** 2020-10

**Authors:** Simon C Langton Hewer, Alan R Smyth, Michaela Brown, Ashley P Jones, Helen Hickey, Dervla Kenna, Deborah Ashby, Alexander Thompson, Paula R Williamson, TORPEDO-CF study group

**Affiliations:** a Department of Paediatric Respiratory Medicine, Bristol Royal Hospital for Children, University of Bristol, Bristol, UK; b Division of Child Health, Obstetrics & Gynaecology, University of Nottingham, Nottingham, UK; c Liverpool Clinical Trials Centre, University of Liverpool, Liverpool Health Partners, Liverpool, UK; d Antimicrobial Resistance and Healthcare Associated Infections Reference Unit, National Infection Service, Public Health England, London, UK; e School of Public Health, Imperial College London, London, UK; f The University of Manchester, Manchester Centre for Health Economics, Manchester, UK

## Abstract

**Background:**

Chronic pulmonary infection with *Pseudomonas aeruginosa* is one of the most important causes of mortality and morbidity in cystic fibrosis. If antibiotics are commenced promptly, infection can be eradicated. The aim of the trial was to compare the effectiveness and safety of intravenous ceftazidime and tobramycin versus oral ciprofloxacin in the eradication of *P aeruginosa.*

**Methods:**

We did a multicentre, parallel group, open-label, randomised controlled trial in 72 cystic fibrosis centres (70 in the UK and two in Italy). Eligible participants were older than 28 days with an isolate of *P aeruginosa* (either the first ever isolate or a new isolate after at least 1 year free of infection). Participants were excluded if the *P aeruginosa* was resistant to, or they had a contraindication to, one or more of the trial antibiotics; if they were already receiving *P aeruginosa* suppressive therapy; if they had received any *P aeruginosa* eradication therapy within the previous 9 months; or if they were pregnant or breastfeeding. We used web-based randomisation to assign patients to 14 days intravenous ceftazidime and tobramycin or 12 weeks oral ciprofloxacin. Both were combined with 12 weeks inhaled colistimethate sodium. Randomisation lists were generated by a statistician, who had no involvement in the trial, using a computer-generated list. Randomisation was stratified by centre and because of the nature of the interventions, blinding was not possible. Our primary outcome was eradication of *P aeruginosa* at 3 months and remaining free of infection to 15 months. Primary analysis used intention to treat (powered for superiority). Safety analysis included patients who received at least one dose of study drug. TORPEDO-CF was registered on the ISRCTN register, ISRCTN02734162, and EudraCT, 2009-012575-10.

**Findings:**

Between Oct 5, 2010, and Jan 27, 2017, 286 patients were randomly assigned to treatment: 137 to intravenous antibiotics and 149 to oral antibiotics. 55 (44%) of 125 participants in the intravenous group and 68 (52%) of 130 participants in the oral group achieved the primary outcome. Participants randomly assigned to the intravenous group were less likely to achieve the primary outcome, although the difference between groups was not statistically significant (relative risk 0·84, 95% CI 0·65–1·09; p=0·18). 11 serious adverse events occurred in ten (8%) of 126 participants in the intravenous antibiotics group and 17 serious adverse events in 12 (8%) of 146 participants in the oral antibiotics group.

**Interpretation:**

Compared with oral therapy, intravenous antibiotics did not achieve sustained eradication of *P aeruginosa* in a greater proportion of patients with cystic fibrosis and was more expensive. Although there were fewer hospitalisations in the intravenous group than the oral group during follow-up, this confers no advantage over oral treatment because intravenous eradication frequently requires hospitalisation. These results do not support the use of intravenous antibiotics to eradicate *P aeruginosa* in cystic fibrosis.

**Funding:**

National Institute for Health Research Health Technology Assessment Programme.

## Introduction

Cystic fibrosis is the most common, life-limiting, autosomal recessive disorder in populations with northern European ancestry and is less commonly reported in other ethnicities.^1^ There are around 30 000 affected individuals in the USA^2^ and 45 000 in Europe.^3^ Cystic fibrosis is a multisystem disorder caused by dysfunction of the cystic fibrosis transmembrane conductance regulator (CFTR) protein.^1^ In the lung, this dysfunction leads to failure of mucociliary clearance, retention of viscid secretions, recurrent infection, and bronchiectasis. Most people with cystic fibrosis die from respiratory failure.^1^ Life expectancy is between 40 years and 50 years.^4^ CFTR modulators, which treat the basic defect of cystic fibrosis, are now available for some patients, dependent on their age and specific mutations;^5^ however, for the foreseeable future, the early treatment of airways infection will remain a core component of cystic fibrosis treatment.

**Research in context**

**Evidence before this study**

Chronic pulmonary infection with *Pseudomonas aeruginosa* is an important cause of mortality and morbidity in cystic fibrosis. In US, UK, and European guidelines, inhaled antibiotics are first-line treatment and intravenous antibiotics second-line treatment for the eradication of early infection with *P aeruginosa*. Our Cochrane systematic review (last updated April 25, 2017) showed that nebulised antibiotics alone or in combination with oral ciprofloxacin are better than no eradication treatment for *P aeruginosa.* We repeated the literature search used in the Cochrane systematic review from Oct 11, 2016, to April 14, 2020, using the same search strategy: relevant trials were identified from the Cochrane Cystic Fibrosis and Genetic Disorders Group Trials Register using the terms “antibiotics” AND (“pseudomonas aeruginosa” OR “mixed infections”) AND (“eradication” OR “unknown”). We also searched the relevant clinical trials databases ClinicalTrials.gov, WHO ICTRP, and ISRCTN using the search terms “cystic fibrosis” AND “pseudomonas aeruginosa” AND “eradication”. Further details, including inclusion criteria and risk of bias assessment, can be found in our Cochrane systematic review. This search revealed a further six publications: two conference abstracts relating to the TORPEDO trial; an infection control study; two references to a study of oral azithromycin for eradication of *P aeruginosa* (as an adjunct to inhaled tobramycin); and a conference abstract relating to a further trial of azithromycin, which was under powered. We therefore conclude that, although intravenous antibiotics are widely used, there are no randomised controlled trials to investigate their effectiveness in eradicating *P aeruginosa* in cystic fibrosis. The TORPEDO-CF trial has been designed to find evidence of superiority of intravenous therapy, if such superiority exists. If intravenous antibiotics are not superior, then guidance can be given that will save health resources.

**Added value of this study**

Our randomised controlled trial is, to our knowledge, the first to compare the effectiveness of intravenous versus oral eradication therapy in children and adults with cystic fibrosis, and showed no significant difference in the number of participants achieving eradication. In the intravenous group, fewer patients were admitted to hospital in the 12 months following eradication. However, this confers no advantage as intravenous eradication usually requires admission whereas oral treatment does not. There was a considerable difference in cost between the two eradication regimens. The study has shown that oral eradication might be used with a cost saving of £5939 (equivalent to 7543 US dollars) per patient. This is the first cost-effectiveness study done as part of a randomised controlled trial of eradication.

**Implications of all the available evidence**

We found that intravenous antibiotics did not achieve eradication of *P aeruginosa* in a greater proportion of children and adults with cystic fibrosis than oral therapy. Intravenous therapy usually requires a prolonged admission to hospital, with consequent family disruption, as well as requiring significant health-care resource. Admission for intravenous therapy in the context of early *P aeruginosa* infection (and without the presence of a significant pulmonary exacerbation), in adults and children with cystic fibrosis, should not be recommended.

Our trial found that, even in the oral group (which had a better primary outcome), only around half of participants were free of infection 15 months after randomisation. Future research should aim to improve rates of eradication through approaches such as earlier detection of *P aeruginosa* and salvage therapy for failed eradication. It should also be a priority to evaluate the effects of CFTR modulators on acquisition and eradication of *P aeruginosa*.

*Pseudomonas aeruginosa* is a highly prevalent pathogen in cystic fibrosis that can be acquired from the environment or from other people with cystic fibrosis.^6^ When airways infection first occurs, the organism is present in planktonic form and is susceptible to antibiotics. In the next state of infection, *P aeruginosa* forms biofilms in the airways, where the organisms are surrounded by exopolysaccharide matrix.^7^ This matrix confers resistance to antibiotics and host defences, and the infection becomes chronic.^8^ The presence of chronic *P aeruginosa* infection in cystic fibrosis is associated with increased mortality and morbidity (including a more rapid decline in lung function and more hospitalisations).^9^ Most people with cystic fibrosis will have chronic airways infection with *P aeruginosa* by their mid-20s.^10^

Current evidence supports starting antibiotic regimens that aim to eradicate *P aeruginosa* as soon as the infection is diagnosed to disrupt nascent biofilms and prevent progression to chronic infection. However, evidence to suggest that any one regimen is better than another is absent.^11^ UK guidelines^12^ recommend an inhaled antibiotic (eg, colistimethate sodium) in combination with a systemic (ie, intravenous or oral) antibiotic because these are thought to be effective against different components of the *P aeruginosa* biofilm.^8^ US and European guidelines favour single agent inhaled tobramycin as first-line treatment.^13^, ^14^ A regimen commencing with 2 weeks of intravenous antibiotics has been suggested to achieve a high eradication rate and is cost-effective;^15^ however, this claim is not supported by data from randomised controlled trials (RCTs). A course of intravenous antibiotics for *P aeruginosa* often involves the cost and inconvenience of a prolonged hospital admission and exposes patients to the risks of drug toxicity—particularly from aminoglycoside antibiotics. “What is the best way of eradicating Pseudomonas aeruginosa in people with cystic fibrosis?” was recently listed as one of the top 10 research priorities for cystic fibrosis in a partnership between people with cystic fibrosis and health-care providers.^16^

Based on the recommendations from a feasibility study,^17^ we designed an RCT to compare an intravenous with an oral regimen for eradication of *P aeruginosa* in children and adults with cystic fibrosis. Both treatment groups also received inhaled colistimethate sodium. We aimed to determine whether one regimen was superior in achieving successful and sustained eradication of *P aeruginosa.*

## Methods

### Study design

TORPEDO-CF was a multicentre, parallel group, open-label RCT, designed to test which of two antibiotic regimens was superior for eradication of *P aeruginosa* in children and adults with cystic fibrosis. The first patient was recruited on Oct 5, 2010, and the last follow-up visit occurred on April 10, 2018. The study was done in 72 cystic fibrosis centres across two countries—70 in the UK and two in Italy.

The London Research Ethics Committee (London, UK) and Etico Regionale Della Liguria (Genoa, Italy) provided ethical approval. An independent data and safety monitoring committee and a trial steering committee provided trial oversight.

There were three major amendments to the protocol during the trial: the follow-up period was defined as 15 months from start of allocated treatment (rather than from randomisation) to avoid bias due to delays in starting intravenous treatment (version 3.0; Sept 1, 2010); increased flexibility in dosing regimens, in line with national clinical guidelines (version 4.0; Dec 13, 2011); and intravenous treatment could be given at home (version 5.0; Jan 11, 2012). Full details of all amendments are given the final trial protocol, version 9.0 (Oct 12, 2016; appendix pp 63–79; HTA monograph). The protocol is also available on the sponsor's website.

### Participants

Eligible participants had a confirmed diagnosis of cystic fibrosis; a recent isolation of *P aeruginosa* from cough swab, sputum (spontaneous or induced), or bronchoalveolar lavage; were aged older than 28 days; and were either *Pseudomonas* naive (ie, never previously isolated *P aeruginosa*) or were *Pseudomonas* free (ie, infection-free for ≥1 year). Participants were required to start allocated treatment within 21 days from the date of the positive microbiology report. Participants were excluded if the *P aeruginosa* was resistant to one or more of the trial antibiotics; if they had a contraindication to any of the trial antibiotics; if they were already receiving *P aeruginosa* suppressive therapy (eg, an inhaled antibiotic); if they had received any *P aeruginosa* eradication therapy within the previous 9 months; or if they were pregnant or breastfeeding. Patients could participate in the TORPEDO-CF trial once only and could not be randomly assigned within 4 weeks of taking part in another intervention trial.

Eligible participants were identified by the principal investigator in each participating centre. Written informed proxy consent was obtained from parents or guardians of children aged younger than 16 years (with assent from the young person) or from the patient themselves if the participant was aged 16 years or older.

### Randomisation and masking

Participants were randomly assigned to either intravenous or oral antibiotic therapy by the principal investigator or a delegated clinician at the site using a secure web-based randomisation system. The randomisation system was controlled centrally by the Liverpool Clinical Trials Centre (University of Liverpool, Liverpool, UK) to ensure allocation concealment, which was maintained by using a secure web based randomisation system. Randomisation lists were generated by a statistician who had no involvement in the trial using a computer-generated list, in a 1:1 ratio, using block randomisation with random variable block length (initial block length of three and then random block sizes of two and four). Randomisation was stratified by centre, but the details were not disclosed to investigators. After entry of participant details, treatment allocation was displayed on a secure webpage and an email confirmation sent to the principal investigator.

### Procedures

Eligible and consenting participants were randomly assigned to receive either 14 days of intravenous ceftazidime at 50 mg/kg three times daily (maximum 3 g per dose) and intravenous tobramycin at 10 mg/kg once daily (maximum 660 mg/day), or 12 weeks treatment with oral ciprofloxacin at 20 mg/kg (maximum 750 mg) twice daily. For children aged younger than 5 years, ciprofloxacin at 15 mg/kg twice daily could be used. Patients in both treatment groups were treated concurrently with 12 weeks of nebulised colistimethate sodium (1 megaunit twice daily for children aged 2 years or younger and 2 megaunits twice daily for children aged older than 2 years and adults). Duration of intravenous, oral, and inhaled therapies was consistent with UK Cystic Fibrosis Trust Guidelines.^12^ Participants who had a subsequent growth of *P aeruginosa* during the trial period were treated as per local guidelines.

A participant's adherence to allocated therapy was monitored using participant-completed treatment diaries. Participants randomly assigned to intravenous antibiotic therapy could have the intravenous component of their regimen in hospital or at home.

Participants were followed up from the time of randomisation to at least 15 months, with trial visits at months 3, 6, 9, 12, 15, 18, 21, and 24. The 3-month visit window was between days 2 and 14 after treatment discontinuation, and the 15 month visit window was between 7 days before the scheduled visit date and 14 days afterwards. Other visits were allowed 7 days either side of the scheduled dates. In participants who were old enough and able to do the procedure, we measured spirometry and collected sputum. Cough swabs were taken from younger children. The initial specimen of *P aeruginosa* and any organisms identified during a recurrent infection were sent to Public Health England's AMRHAI Reference Unit (Colindale, UK) for strain typing by variable number tandem repeat (VNTR) analysis at nine loci.^18^

### Outcomes

The primary outcome was eradication of *P aeruginosa* from respiratory samples (cough swab, sputum, or bronchoalveolar lavage) at 3 months from commencing trial treatment and remaining free of infection to 15 months. We chose 12 months, following completion of eradication, as a reasonable period to evaluate whether eradication was sustained. Some participants did not have samples collected during the pre-specified protocol windows for the primary outcome at 3 months and 15 months. It was therefore decided, during a blind data review after trial completion, to extend the window at which the 3 month sample could be taken to 4 weeks either side of the expected visit date, providing treatment had ceased at least 48 h before sampling. Similarly, the window was extended to four weeks either side of the expected 15 month visit date. This decision was made to avoid excluding patients whose samples were taken outside the protocol-specified window. Secondary outcomes that utilised these sample results included time to recurrence of the original *P aeruginosa* infection and re-infection with a different genotype of *P aeruginosa* or other respiratory organisms. To avoid excessive participant burden, specimens for microbiology were collected at routine clinic visits. Data on secondary outcomes, including forced expiratory volume in 1 s (FEV_1_), forced vital capacity (FVC), forced expiratory flow in mid expiration (FEF_25–75_), oxygen saturation, height, weight, body-mass index (BMI), number of pulmonary exacerbations,^19^ number of hospital admissions, number of days spent as an inpatient, carer and participant burden, were collected at baseline and at all of the routine trial visits.

Quality of life (QOL) and health-related QOL instruments, CFQ-R^20^ and EQ-5D-3L^21^ respectively, were completed by the participant, carer, or both (dependent on age) and collected at baseline and then at months 3, 15, and 24. The EQ-5D-3L is a generic measure frequently used to measure health status and can be used to calculate quality-adjusted life-years (QALYs).^22^ Despite the EQ-5D-3L being designed primarily for adults, it is still advocated for use in children for reasons of comparability.^23^ Bespoke health service use diaries were collected (UK sites only) at baseline (completed retrospectively) and months 3, 6, 9, 12, and 15 (completed prospectively) to calculate cost per patient (from the National Health Service and Personal Social Services perspective) and assess incremental cost-effectiveness.

An assessment of adverse events was undertaken at each trial visit until 28 days after treatment cessation. All serious adverse events were recorded but only those serious events where the causal relationship to the trial treatment had been assessed and judged by the principal investigator to be possibly, probably, or almost certainly related to the trial treatment and had occurred within the reporting timeframe were expedited.

### Statistical analysis

To inform the sample size calculation, data on the number of patients with successful eradication of *P aeruginosa* 3 months from the start of treatment and remaining free of infection to 15 months were obtained from an audit of all current cystic fibrosis patients at Alder Hey Children's Hospital (Liverpool, UK). Data were included for treatments given from 1994–2007 treated according to standard UK CF Trust Guidelines.^12^ Data on 48 children were available. Infection was eradicated at 3 months in 37 (77%) and 28 (58%) of 48 children remained infection-free to 15 months. For 90% power at a 5% level of significance (two-sided), to detect a difference between groups of 20% (between 55% and 75% successful eradication), 128 participants were required in each group. A 20% difference between the two treatment regimens was deemed to be of clinical importance, since the more intensive intravenous treatment would need to be justified by a substantial benefit. We estimated that 10% of participants in each treatment group would not provide primary outcome data giving a recruitment target of 286 participants.

No formal interim analysis was planned, but analyses of the accumulating data on recruitment, protocol deviations, baseline characteristics, adherence, withdrawals, missing data, and safety data were performed annually for review by the independent data and safety monitoring committee. No member of the trial team, apart from the nominated statisticians, had access to these data.

All analyses were pre-specified in a statistical analysis plan.^24^ Evaluation of clinical effectiveness followed the principle of intention to treat (ITT) as far as was practically possible. We analysed safety in patients who received at least one dose of their allocated trial medication (the safety population). Analyses were performed using SAS, version 9.3, or later (North Carolina, USA).

For the primary outcome we calculated the relative risk (RR) and 95% CI, along with a two-sided p value from a χ^2^ test. Several sensitivity analyses were prespecified to test the robustness of this analysis. For time to recurrence of the original *P aeruginosa* infection, we analysed data using the log-rank test, presenting the data as a Kaplan-Meier plot, with a Cox proportional hazards model adjusted for baseline variables used as appropriate to calculate the hazard ratio and 95% CI. We used a repeated measures random effects model to analyse spirometric data (Quanjer GLI-2012 regression equations),^25^ oxygen saturation, Z scores for anthropometric data (children), BMI (adults), and QOL data. The dependent variables were the post baseline values and covariates were baseline values, treatment, time of follow-up measurement, and treatment by time of follow-up measurement interaction. The mean differences between the two treatment groups at 15 months were calculated, presented with 95% CIs and p values. Pulmonary exacerbations were as defined by Rosenfeld and colleagues.^19^ We compared the number of exacerbations per participant in the 15 months from randomisation, between study groups, using the Mann-Whitney test. We compared the number of inpatient days in each group during the treatment phase and the number of inpatient days during the 12 months after eradication treatment was complete using the Mann-Whitney test. In the intravenous group, we excluded the number of days of intravenous antibiotics given in hospital (as mandated by the protocol). We compared the proportion of patients who were admitted to hospital at least once with a χ^2^ test between groups.

### Economic analysis

A prospective economic evaluation was done alongside the RCT to assess the cost-effectiveness of oral versus intravenous antibiotic therapy. The primary analysis used a National Health Service and Personal Social Services perspective for the collection and incorporation of resource use. In the oral versus the intravenous therapy groups, we compared the incremental cost per successful *P aeruginosa* infection eradication from 3 months through to 15 months post randomisation. The time horizon for the analysis was 15 months post randomisation.

In the secondary analysis, utility values and survey timepoints were used to generate QALYs using the area under the curve method. Utilities were calculated from the EQ-5D-3L questionnaire, completed by patients or by proxy (carers), by using societal preference weights.^21^ QALYs were calculated based on both 15-month and 24-month horizons. Secondary analysis measured the incremental net monetary benefit (INMB) of treating patients with oral versus intravenous antibiotics within a cost-utility analysis. The INMB was calculated by subtracting the change in costs (oral *vs* intravenous) from the change in QALYs, valued at the £20 000 per QALY threshold, as typically used by the National Institute for Health and Care Excellence (NICE).^26^ A positive INMB implies that oral treatment is more cost-effective than intravenous treatment.^27^ Sensitivity analyses explored key structural uncertainties in the analysis identified a priori including the use of the specialised cystic fibrosis reimbursement tariff for patients, rather than activity-based costing, as well as also including societal costs.

Total resource use and costs for each patient within the clinical trial were calculated. All costs were calculated in GBP using the price year 2016/17 (appendix pp 1–5). Where possible, unit costs were sourced from national databases (appendix pp 1–5). Missing resource utilisation data and utility values were multiply imputed (appendix pp 22, 26). Costs or outcomes beyond 12 months were not discounted because of the short time horizon of the trial. Regression analysis for incremental costs and outcomes were adjusted for baseline utility and age because of the marked range of ages in this study. To account for statistical uncertainty and the correlation between costs and patient outcomes, the data were bootstrap sampled with replacement 2000 times.

TORPEDO-CF was registered with ISRCTN, ISRCTN02734162, and EudraCT, 2009-012575-10.

### Role of the funding source

The trial was conceived after a feasibility study^17^ commissioned by the funder, the UK National Institute for Health Research (NIHR) Health Technology Assessment Programme. For TORPEDO-CF, the funder of the study had no role in data collection, data analysis, data interpretation, or writing of the Article. The corresponding author had full access to all the study data and had final responsibility for the decision to submit for publication.

## Results

**Figure 1:**
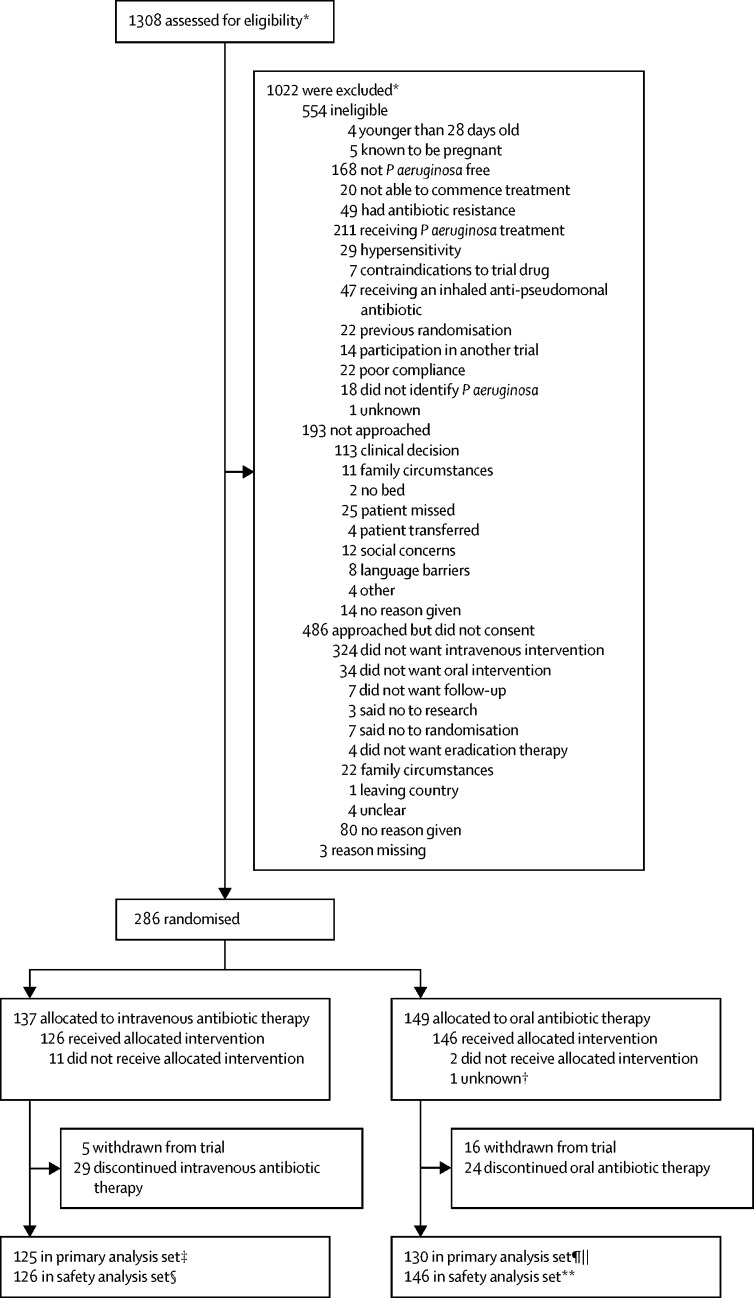
Trial profile *Multiple screenings were allowed; therefore, the number of reasons is greater than the number of patients excluded. †Patient randomly assigned but all data completely removed because sign off from the principal investigator could not be obtained. ‡12 excluded from primary analysis set because no sample at 15-month window. §11 excluded from safety analysis set because they did not receive any allocated treatment. ¶18 excluded from primary analysis set because no sample at 15-month window. ||Patient was randomly assigned, but all data were removed because principal investigator sign off could not be obtained. **Two excluded from safety analysis set because they did not receive any allocated treatment.

The first participant was randomly assigned on Oct 5, 2010, and the final participant on Jan 27, 2017. 1308 patients from 72 centres were screened for eligibility; 1022 did not meet the eligibility criteria (figure 1). 286 patients from 61 centres (range 1–22 patients per site) were randomly assigned to treatment: 137 to intravenous antibiotic therapy and 149 to oral antibiotic therapy. 285 participants were included in the ITT analysis. One patient was excluded after randomisation because principal investigator approval was not provided at database lock and therefore the participant's data could not be used. Of 137 participants in the intravenous group, 11 (8%) did not receive treatment (neither allocated intervention nor colistimethate sodium) and 29 (21%) stopped treatment early. In the oral group, two (1%) of 148 participants did not start treatment and 24 (16%) stopped treatment early. The most common single reason for not completing allocated treatment in the intravenous group was difficulty with intravenous access (12 [30%] of 40), and an adverse event was the most common reason in the oral group (13 [50%] of 26).

Baseline participant characteristics in each group are shown in table 1. A greater, but not significant, number of children aged 28 days to 23 months were randomly assigned to the intravenous group (42 [31%] of 137) than the oral group (28 [19%] of 146), but there were no other notable imbalances in baseline characteristics. None of the baseline samples that confirmed initial infection with *P aeruginosa* were resistant to any of the study drugs. Randomisation was stratified to ensure approximately equal numbers of participants in each group within a centre. Due to some sites recruiting small numbers and an initial block size of three participants, fewer participants were allocated to the intravenous group. Only 15 (5%) participants were adults (ie, aged ≥18 years).

**Table 1:** Baseline characteristics of the intention-to-treat population

		**Intravenous antibiotic therapy (n=137)**	**Oral antibiotic therapy (n=148)**
Age group*			
	Infants and toddlers (28 days to 23 months)	42 (31%)	28 (19%)
	Children (2 to 11 years)	71 (52%)	92 (62%)
	Adolescents (12 to 17 years)	18 (13%)	19 (13%)
	Adults (18 to 64 years)	6 (4%)	9 (6%)
Sex			
	Male	63 (46%)	67 (45%)
	Female	74 (54%)	81 (55%)
*Pseudomonas aeruginosa*			
	Naive	81 (59%)	93 (63%)
	Free	56 (41%)	55 (37%)
*Pseudomonas aeruginosa* confirmed by			
	Bronchoscopy lavage	0	3 (2%)
	Cough plate	5 (4%)	4 (3%)
	Cough swab	91 (66%)	91 (61%)
	Cough swab and sputum sample	0	1 (1%)
	Cough swab and throat swab	0	1 (1%)
	Oropharyngeal aspirate	1 (1%)	0
	Nasopharyngeal aspirate	0	1 (1%)
	Sputum sample (not induced)	34 (25%)	39 (26%)
	Sputum sample (induced)	3 (2%)	5 (3%)
	Throat swab	2 (1%)	3 (2%)
	Unknown	1 (1%)	0
Other micro-organisms detected			
	Candida albicans	11 (8%)	17 (11%)
	MRSA†	0	2 (1%)
	*Burkholderia cepacia* complex	0	0
	Aspergillus fumigatus	2 (1%)	2 (1%)
	Other organisms	26 (19%)	31 (21%)
Genotype			
	p·Phe508del and p·Phe508del	70 (51%)	90 (61%)
	p·Phe508del and other	40 (29%)	43 (29%)
	p·Phe508del and unknown	4 (3%)	5 (3%)
	Other and other	12 (9%)	7 (5%)
	Unknown	11 (8%)	3 (2%)
Pulmonary exacerbation^20^ present		18 (13%)	17 (11%)
BMI Z score (paediatric)†		n=125; 0·3 (1)	n=131; 0·3 (0·9)
BMI (adults; m/kg^2^)†		n=6; 24·6 (1·8)	n=9; 23·2 (2·3)
Time from *P aeruginosa* isolation to treatment initiation (days)†		n=126; 9 (5)	n=145; 7 (5)
FEV_1_percentage predicted (l)†		n=67; 86·6 (15·8)	n=70; 85·7 (16)
FVC percentage predicted (l)†		n=67; 92·2 (15·5)	n=70; 95·1 (14·5)
FEF_25–75_ %predicted (l)†		n=44; 72·7 (26·6)	n=53; 70·6 (30·3)
O_2_ saturation (%)†		n=118; 97·7 (1·4%)	n=133; 97·7 (1·7%)

Data are n (%), mean (SD) or n/N (%). MRSA=methicillin resistant *Staphylococcus aureus*. BMI=body-mass index.

* Date of birth was not provided for two participants to allow age to be calculated. Age at randomisation was provided after database lock and analysis and added to the baseline table manually.

† Data not available for all randomly assigned patients.

Participants in the intravenous group received a mean of 11·5 days (SD 5·3) of ceftazidime and 11·0 days (5·1) of tobramycin (protocol-specified duration 10–14 days) based on data collected from 137 participants. Of the 126 participants who received at least one dose of intravenous treatment, 67 (53%) received all doses in hospital and 58 (46%) received some treatment at home (data were missing for one participant). In the oral group, the protocol specified 168 doses of ciprofloxacin over 84 days and the mean adherence rate was 93·2% (SD 17·3) based on data from 105 participants who returned fully completed treatment diaries. The protocol specified 84 days of colistimethate sodium for both groups. The mean adherence rate (over the first 84 days) was 82·1% (31·5) in the intravenous (n=97) and 91·6% (19·0) in the oral group (n=105).

We excluded 30 participants from the primary analysis (12 intravenous and 18 oral) in whom *P aeruginosa* was not detected after completion of eradication, but who did not have a sample taken at the 15 month time window. The primary outcome (successful eradication at 3 months and remaining infection free through to 15 months) was achieved by 55 (44%) of 125 participants in the intravenous and 68 (52%) of 130 participants in the oral group. Participants who were randomly assigned to the intravenous group had a reduced chance of having successful eradication of *P aeruginosa* 3 months after the start of treatment and remaining infection free through 15 months, although this was not statistically significant (risk difference −0·08, 95% CI −0·21 to 0·04; relative risk [RR] 0·84, 95% CI 0·65 to 1·09; p=0·18). The types of samples that confirmed participants had a recurrence of *P aeruginosa* (post hoc) and the number of these samples that were resistant to any of the study drugs (post hoc) are provided in the appendix (p 6).

**Figure 2:**
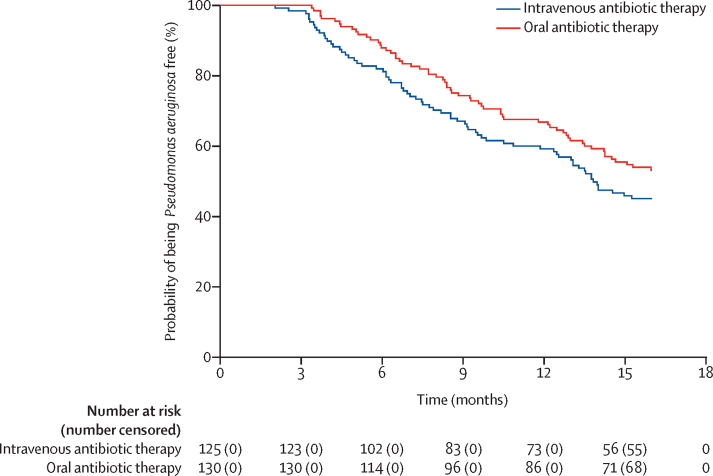
Kaplan-Meier plot

Five sensitivity analyses confirmed the conclusion of the primary analysis (p 7). An additional sensitivity analysis, where a logistic regression was fitted including centre as a random effect, confirmed centre had no effect on the primary results (p=0·22). A post-hoc analysis of time to *P aeruginosa* isolation found that *P aeruginosa* recurrence occurred sooner in the intravenous than the oral group, but this was not statistically significant (hazard ratio 1·31; 95% CI 0·93 to 1·85; p=0·12; figure 2). Two post-hoc analyses were done comparing the proportion of participants in whom infection was successfully eradicated by 3 months and subgroup analyses of patients who were *P aeruginosa* naive and *P aeruginosa* free. The first post-hoc analysis showed no significant difference between treatment groups (RR 0·92, 95% CI 0·85 to 1·00; p=0·04). In participants who were *P aeruginosa* free, the primary outcome was achieved by 23 (47%) of 49 patients in the intravenous and 21 (42%) of 50 patients in the oral group. Participants who were *P aeruginosa* free and randomly assigned to the intravenous group had an increased chance of having successful eradication of *P aeruginosa* 3 months after the start of treatment and remaining infection-free through 15 months compared with the oral treatment group, although this was not statistically significant (risk difference 0·05, 95% CI −0·15 to 0·24; RR 1·12, 95% CI 0·72 to 1·74). In participants who were *P aeruginosa* naive, the primary outcome was achieved by 32 (42%) of 76 patients in the intravenous group and 47 (59%) of 80 patients in the oral group. Participants who were *P aeruginosa* naive and randomly assigned to the intravenous group had a reduced chance of having successful eradication of *P aeruginosa* 3 months after the start of treatment and remaining infection free through 15 months compared with the oral treatment group (risk difference −0·17, 95% CI −0·32 to −0·01; RR 0·72, 95% CI 0·53 to 0·99). A Mantel-Haenszel test for interaction was done and the relative risks were not significantly different between the two subgroups (p=0·19).

Samples were available for VNTR typing at baseline and *P aeruginosa* infection recurrence was noted for 42 participants (25 in the intravenous and 17 in the oral group). Using this method, 19 (76%) of 25 participants in the intravenous group and 12 (71%) of 17 participants in the oral group had the same strain of *P aeruginosa* at baseline and recurrence. Because of the small number of participants included, caution is required in interpreting the analysis of the time to recurrence of the original *P aeruginosa* infection (appendix p 8) and the analyses of re-infection with a different and distinct genotype of *P aeruginosa* (appendix p 9). We assessed the effect of the treatment regimen on various measures of lung function (in participants aged 5 years and older) and found no evidence of an effect over time on percentage predicted FEV_1_ (estimated mean difference for intravenous compared with oral treatment 2·08, 95% CI −0·99 to 5·14; p=0·18), percentage predicted FEF_25–75_ (3·46, −3·74 to 10·66; p=0·35), or oxygen saturation (0·05, −0·20 to 0·29; p=0·71). FVC was significantly better in the intravenous group than the oral group (3·14, 0·15 to 6·14; p=0·04); however, this finding should be interpreted with caution because there was no adjustment for multiple testing (table 2). Similarly, BMI (adults) was significantly lower in the intravenous group than the oral group (–0·73, −1·39 to −0·08; p=0·029), but this finding was based on a small number of adults with available data (n=13). There was no evidence of an effect at 15 months on oxygen saturation or on height for age Z score, weight for age Z score, or BMI Z scores in children (table 2).

**Table 2:** Estimates from random effects models for longitudinal secondary outcome measures at 15 months

	**Intravenous antibiotic therapy**	**Oral antibiotic therapy**	**Estimated mean difference (95% CI)**	**p value**
Percentage predicted FEV_1_	86·19 (1·10)	84·11 (1·10)	2·08 (−0·99 to 5·14)	0·18
Percentage predicted FVC	94·08 (1·08)	90·94 (1·08)	3·14 (0·15 to 6·14)	0·04
Percentage predicted FEF_25–75_	72·39 (2·73)	68·93 (2·45)	3·46 (−3·74 to 10·66)	0·35
Oxygen saturation	97·83 (0·09)	97·78 (0·09)	0·05 (−0·20 to 0·29)	0·7
Weight for age Z score	0·11 (0·05)	0·13 (0·05)	−0·02 (−0·15 to 0·11)	0·79
Height for age Z score	−0·34 (0·04)	−0·31 (0·04)	−0·03 (−0·13 to 0·07)	0·57
BMI Z score (paediatric)	0·34 (0·05)	0·33 (0·05)	0·01 (−0·14 to 0·16)	0·91
BMI (kg/m^2^; adult)	23·51 (0·22)	24·25 (0·24)	−0·73 (−1·39 to −0·08)	0·029

Data are mean (SE) estimated from model, unless otherwise indicated. Random effect model contains: baseline value, treatment group, time of follow-up measurement, and treatment group by time of follow-up measurement interaction term as independent variables in the model. BMI=body mass index.

During the 15 month follow-up, 52 (36%) of 146 participants in the oral antibiotic group and 38 (28%) of 137 in the intravenous group had a pulmonary exacerbation. The difference was not statistically significant (RR 0·78, 95% CI 0·55–1·10; p=0·16). Significantly fewer participants in the intravenous group (40 [31%] of 129) than the oral group (61 [45%] of 136) were admitted to hospital in the 12 months after eradication treatment (RR 0·69, 95% CI 0·50 to 0·95; p=0·020). During the 3 month treatment phase, the median hospital stay for participants in both groups was 0 days (range 0–29 for intravenous [n=135] and 0–15 for oral [n=143]; p=0·066). During the 12-month post-treatment period, the median hospital stay was also 0 days for both groups (range 0–69 for intravenous [n=129] and 0–64 for oral [n=136]; p=0·005). There were no statistically significant differences between study groups for the number of participants who isolated Methicillin-resistant *Staphylococcus aureus* (MRSA), *Burkholderia cepacia* complex, *Aspergillus* spp, or *Candida* spp (table 3). The number of participants who grew other organisms of interest is reported in the appendix (p 10). No planned statistical analyses were done for these organisms.

**Table 3:** Other sputum or cough microbiology over 15 months after randomisation

	**Intravenous antibiotic therapy**	**Oral antibiotic therapy**	**Relative risk (95% CI)**	**p value**
Methicillin-resistant *Staphylococcus aureus*	4/135 (3%)	2/140 (1%)	2·07 (0·39–11·14)	0·44
*Burkholderia cepacia* Complex	2/135 (1%)	4/139 (3%)	0·51 (0·10–2·76)	0·68
*Candida* spp	55/136 (40%)	55/142 (39%)	1·04 (0·78–1·40)	0·77
*Aspergillus* spp	14/135 (10%)	20/139 (14%)	0·72 (0·38–1·37)	0·31

Data are n/N (%), unless otherwise indicated.

The QOL questionnaire was only completed for or by participants aged 6 years and older (134 of 285 randomly assigned patients; intravenous group n=62, oral group n=72). 106 participants were included in the analysis of the majority of domains in the self-report questionnaire (some domains included 105 participants because of missing data). There were no statistically significantly differences between the two treatment groups at 15 months across any of the domains in each questionnaire (appendix p 11). The median number of days of absenteeism from education or work for carers and for participants was not statistically significantly between the two treatment groups (appendix p 12).

126 non-serious adverse events were reported from 60 (48%) participants in the intravenous group, and 136 non-serious adverse events from 72 (49%) participants in the oral group. The three most common adverse events (>5% of participants in either group) were cough, upper respiratory tract infection, and productive cough (table 4). One adverse event (malignant melanoma), which was classified as severe, was deemed to be unrelated to the trial medication. Summary data for all non-serious adverse events are detailed in the appendix (p 13). 11 serious adverse events were reported from ten (8%) participants in the intravenous group and 17 serious adverse events reported from 12 (8%) participants in the oral antibiotic group (table 5). No participants died or had any suspected unexpected serious adverse reactions.

**Table 4:** Most common non-serious adverse events

		**Intravenous antibiotic therapy (n=126)**		**Oral antibiotic therapy (n=146)**		**Total (n=272)**	
		Events	Participants	Events	Participants	Events	Participants
Any non-serious adverse event*		126	60 (48%)	136	72 (49%)	262	132 (49%)
Most common non-serious adverse events†							
	Cough	26	22 (17%)	28	23 (16%)	54	45 (17%)
	Upper respiratory tract infection	15	11 (9%)	3	2 (1%)	18	13 (5%)
	Productive cough	5	5 (4%)	8	8 (5%)	13	13 (5%)

Data are n or n (%).

* Nine additional adverse events were reported by nine participants in the intravenous antibiotic therapy group and five adverse events from five participants in the oral antibiotic therapy group but have not been included here as they were the event of interest for the primary outcome (*P aeruginosa* isolation) so should not have been reported as an adverse event. Three additional adverse events were reported by three participants in the oral antibiotic therapy group but have not been included here as they contributed to the analysis of the outcome number of pulmonary exacerbations.

† Reported by at least 5% of participants in either treatment group.

**Table 5:** Serious adverse events, according to trial group

	**Intravenous antibiotic therapy (n=126)**		**Oral antibiotic therapy (n=146)**		**Total (n=272)**	
	Events	Participants	Events	Participants	Events	Participants
Gastrointestinal disorders	2	2 (2%)	1	1 (1%)	3	3 (1%)
General disorders and administration site conditions	1	1 (1%)	2	2 (1%)	3	3 (1%)
Hepatobiliary disorders	1	1 (1%)	0	0	1	1 (<1%)
Infections and infestations*	1	1 (1%)	7	6 (4%)	8	7 (3%)
Nervous system disorders	1	1 (1%)	0	0	1	1 (<1%)
Psychiatric disorders	0	0	1	1 (1%)	1	1 (<1%)
Renal and urinary disorders	1	1 (1%)	0	0	1	1 (<1%)
Respiratory, thoracic, and mediastinal disorders	0	0	6	5 (3%)	6	5 (2%)
Skin and subcutaneous tissue disorders	1	1 (1%)	0	0	1	1 (<1%)
Surgical and medical procedures	1	1 (1%)	0	0	1	1 (<1%)
Vascular disorders	2	2 (2%)	0	0	2	2 (1%)
Total*	11	10 (8%)	17	12 (8%)	28	22 (8%)

Data are n or n (%).

* Three additional serious adverse events were reported by three participants in the oral antibiotic therapy group but have not been included here as they were the event of interest for the primary outcome (*P aeruginosa* isolation) so should not have been reported as a serious adverse event. One additional adverse event was reported by one participant in the oral antibiotic therapy group but has not been included here as it contributed to the analysis of the outcome number of pulmonary exacerbations.

In the economic evaluation, before adjusting for covariates, the oral group had larger follow-up costs (excluding intervention costs) than the intravenous group, predominantly reflecting greater costs associated with follow-up inpatient stays (appendix pp 16–18), with all other costs and resource use being similar between the groups (appendix pp 19–22). Patient health-related QOL, without adjusting for covariates, was similar across the two groups for each timepoint (appendix p 22).

Incremental total costs, outcomes, and cost-effectiveness for the primary and secondary analyses, adjusted by baseline characteristics, are shown in table 6. Overall, oral treatment was less costly than intravenous treatment when including intervention costs, with the incremental difference in mean costs being –£5939 (95% CI −7107 to −4666) after adjusting for baseline covariates. Much of the overall total cost difference reflects the inpatient stay cost for intravenous therapy. In sensitivity analyses, where specialised cystic fibrosis tariffs were used to cost therapy and hospitalisations, the incremental difference in costs was lower, at –£653 (–1198 to −80) for oral treatment versus intravenous treatment (appendix p 23), reflecting the higher banding placed on reimbursement for follow-up hospitalisations, which were greater in the oral group (appendix p 5). For a 15 month time horizon, patients on oral treatment gained 0·035 QALYs (95% CI −0·007 to 0·088) versus patients receiving intravenous treatment. After 24 months, patients on oral treatment gained 0·058 QALYs (–0·004 to 0·140) for oral versus intravenous treatment.

**Table 6:** Incremental costs, outcomes, ICERs, and incremental net benefit between trial groups (oral *vs* intravenous)

	Incremental cost (£)*	Incremental outcome*	**ICER**	**Incremental net monetary benefit (£)**†
Primary analysis: percentage successful eradication, NHS and PSS perspective costs, 15-month horizon, adjusted	−5938·5 (95% CI −7107·4 to −4666·3)	0·091 (95% CI −0·034 to 0·22)	Oral dominates‡	NA§
Secondary analysis: 15-month horizon QALYs, NHS, and PSS perspective costs, covariate	−5938·5 (95% CI 7107·4 to −4666·3)	0·035 (95% CI −0·007 to 0·088)	Oral dominates‡	6770·8 (95% CI 5027·4 to 7906·2)

ICER=Incremental Cost Effectiveness Ratio. NHS=National Health Service. PSS=Personal Social Services. NA=not applicable. QALY=quality-adjusted life-years

* Adjusting for baseline EQ5D and age (in days) in linear regression.

† At a cost-effectiveness threshold of £20 000 per QALY.

‡ Dominates (ie, more effective and less costly).

§ There is no societally accepted willingness-to-pay threshold associated with the primary outcome measure and so this is not reported.

In all scenarios, including sensitivity analyses (appendix p 23), oral treatment led to reductions in costs versus intravenous treatment and was also more effective (oral dominates intravenous). For a cost-effectiveness threshold of £20 000 per QALY, oral treatment generated £6771 (95% CI 5027 to 7906) benefit per patient versus intravenous treatment. Cost-effectiveness planes and acceptability curves (appendix pp 24–25) show that the results were robust dependent on the threshold used for cost-effectiveness.

## Discussion

We tested the hypothesis that an intravenous antibiotic regimen is more effective than oral treatment for eradicating *P aeruginosa* in people with cystic fibrosis. Inhaled colistimethate sodium was included in both regimens. Intravenous treatment was not superior in achieving the primary outcome, eradication of *P aeruginosa* at 3 months and remaining free of infection to 15 months. In the 12 months after completion of the eradication regimen, significantly fewer patients in the intravenous group were admitted to hospital than in the oral group. This difference might be because patients who have already had one hospital admission for eradication are less likely to accept a further admission in the subsequent 12 months. Health-related QOL was similar in the two groups, suggesting 2 weeks of intravenous treatment did not have a negative effect.

A Cochrane systematic review^11^ has shown that eradication of *P aeruginosa* with an antibiotic eradication regimen in patients with cystic fibrosis is effective; however, no evidence exists favouring one eradication regimen over another. UK guidelines recommend inhaled colistimethate sodium and oral ciprofloxacin as first-line treatment.^12^ Guidelines from the USA^13^ and Europe^14^ favour inhaled tobramycin as a single agent, but acknowledge that other regimens (including colistimethate sodium and ciprofloxacin) can be used. Our trial has shown that there is no advantage in choosing intravenous treatment as first-line treatment. When a regimen comprising inhaled colistimethate sodium and oral ciprofloxacin was compared with inhaled tobramycin, there was no difference in eradication rates,^28^ suggesting inhaled tobramycin would perform equally well against intravenous treatment. The eradication rates achieved in our trial (44% intravenous and 52% oral) are less than those achieved in the ELITE trial^29^ of 28 days versus 56 days of inhaled tobramycin (66–69% eradication at 27 months). This might reflect the exclusion of pseudomonas antibody-positive patients from the ELITE trial.^29^ Our trial evaluating a single course of eradication treatment is not directly comparable with trials evaluating regular, cycled therapy such as the EPIC trial.^30^ Eradication treatment should be initiated as soon as possible after *P aeruginosa* is detected in respiratory secretions.^31^ A recent crossover trial^32^ of eradication in young children showed a much lower eradication rate in those who had placebo in the initial 28 day treatment period. In the TORPEDO-CF trial, the protocol mandated that the eradication regimen should start not more than 21 days after *P aeruginosa* isolation.

Our health economic evaluation showed a cost saving of £5939 (equivalent to US$7543) per patient with the use of oral versus intravenous treatment. The economic evaluation found that oral eradication therapy was likely to have similar effectiveness for the primary clinical measure, but that oral eradication was considerably cheaper. Consequently, there was high certainty that oral treatment was more cost-effective than intravenous therapy. In secondary analysis, using the EQ-5D-3L to calculate QALYs (commonly used for health technology assessment), oral therapy was also highly likely to be cost-effective versus intravenous therapy. However, estimates on QALYs and utility values reported in this study can only be considered indicative as EQ-5D-3L scores from proxy and self-reported instruments were combined.^33^

The TORPEDO-CF trial used a pragmatic design to minimise the burden of participation. Respiratory specimens for microbiology were collected at routine clinic visits, which meant that not all were obtained in the 3 week window at the end of the 15 month follow-up. To address this issue we extended the window to include patients with samples 4 weeks either side of 15 months and did a sensitivity analysis using the next sample collected after the 15 month window. We collected sputum or a cough swab when collection of sputum was not possible. We chose a minimum clinically important difference for the primary outcome of 20%. A difference of this size is plausible. In the largest trial^30^ of *P aeruginosa* eradication to date, a difference of 17% was seen in the rates of eradication between treatment groups. Discussions with the patient community informed the design of the TORPEDO-CF trial and these discussions indicated that, if intravenous treatment was more effective, the magnitude of this effect should be sufficient to justify the inconvenience of hospital admission for intravenous antibiotics.

A strength of our study was the large sample size and the length of follow-up. In the first generation of placebo-controlled eradication trials, sample size was often small and microbiological eradication was reported immediately after the end of eradication treatment.^34^ In 2012, a large RCT evaluating two 28 day eradication regimens showed that more than 60% of participants had further infection with *P aeruginosa* during a median follow-up of 16 months.^35^ Arguably, with longer follow-up, recurrence of *P aeruginosa* will be influenced to a lesser degree by a single course of eradication treatment. Hence, the 12 months of post-eradication follow-up used in our study is reasonable.

Feasibility data suggested that 25% of eligible patients would be adults.^17^ However, we recruited only 15 adults out of a total sample size of 286 participants, because only a small number of adult centres participated. We therefore advise caution in applying these trial findings to the adult cystic fibrosis population. Many patients and families had a strong preference for one eradication regimen: 324 declined to participate because they did not want intravenous therapy and 34 because they did not want an oral regimen. Our feasibility study suggested that 45% of parents and patients would consider participation. The consent rates achieved in TORPEDO-CF were lower, at 286 (37%) of 772 eligible patients approached.

Patient engagement work identified *P aeruginosa* eradication (UK)^16^ and microorganism detection and treatment (USA)^36^ as important research priorities—both prioritised reducing treatment burden. Future studies should combine long-term follow-up with regimens to reduce recurrence after eradication. The 2018 OPTIMIZE trial^37^ used 18 months of oral azithromycin as an adjunct to eradication with inhaled tobramycin, but found no difference in time to recurrence. When a treatment (such as intravenous antibiotics) is burdensome, but no more effective, it follows that patients should be offered an eradication regimen that is appropriate to their clinical condition and personal circumstances. *P aeruginosa* might be identified at the time of a pulmonary exacerbation when intravenous antibiotics are clinically indicated. In other cases, when the patient is asymptomatic, oral eradication is appropriate. In our trial, only eight (13%) of 137 of patients in the intravenous group and 17 (11%) of 148 patients in the oral group had an exacerbation at baseline.

Intravenous treatment often entails hospital admission, requires intravenous access (which might be traumatic),^38^ and carries the risk of side-effects, including nephrotoxicity and ototoxicity with aminoglycosides such as tobramycin.^39^ Because there were no important clinical benefits to the use of intravenous over oral therapy, the large difference in cost suggests that oral therapy should usually be recommended for eradication of early infection with *P aeruginosa* in cystic fibrosis. If the findings of this trial are implemented in routine clinical practice, most patients will receive oral eradication treatment as an outpatient and many hospital admissions will be avoided, which in turn will reduce both treatment burden and health-care costs.

## Data sharing

Anonymised data collected during this trial and any additional documents will be available to access. Proposals should be directed to SCLH, on behalf of the TORPEDO-CF Trial Management Group (simon.langtonhewer@bristol.ac.uk). Access will be provided to researchers after the proposal has been reviewed and agreed by the trial data sharing committee.
