# Supplementary material for: Intravenous versus oral antibiotics for eradication of Pseudomonas aeruginosa in cystic fibrosis (TORPEDO-CF): a randomised controlled trial
Source: Lancet Respir Med. 2020 Oct;8(10):975–86. doi: 10.1016/S2213-2600(20)30331-3 (PMC7606906; doi:10.1016/S2213-2600(20)30331-3)
Supplement: Supplementary appendix [file mmc1.pdf]

THE LANCET
Respiratory Medicine

Supplementary appendix

This appendix formed part of the original submission and has been peer reviewed. We post it as supplied by the authors.

Supplement to: Hewer SCL, Smyth AR, Brown M, et al. Intravenous versus oral antibiotics for eradication of *Pseudomonas aeruginosa* in cystic fibrosis (TORPEDO-CF): a randomised controlled trial. *Lancet Respir Med* 2020; **8:** 975–86.

TORPEDO-CF Study Group participating centres and investigators

Participating Centre	Investigators
Addenbrooke's Hospital – Paediatrics	Dr Robert Ross-Russell
Alder Hey Children's Hospital - Paediatrics	Professor Kevin Southern
Barts and The London Children's Hospital - Paediatrics	Dr Chinedu Nwokoro
Belfast City Hospital - Adults	Dr Damian Downey
Birmingham Children's Hospital - Paediatrics	Dr Maya Desai
Birmingham Heartlands Hospital - Adults	Dr Joanna L. Whitehouse
Birmingham Heartlands Hospital - Paediatrics	Dr Sarah Denniston
Bradford Royal Infirmary - Paediatrics	Dr Eduardo Moya
Bristol Royal Hospital for Children - Paediatrics	Dr Simon Langton Hewer
Bristol Royal Infirmary - Adults	Dr Simon Langton Hewer
Chesterfield Royal Hospital - Paediatrics	Professor Jim Crossley
Conquest Hospital - Paediatrics	Dr Geeta Gopal
Countess of Chester Hospital - Paediatrics	Dr Ravi Jayaram
Darlington Memorial Hospital - Paediatrics	Dr John Furness
Derriford Hospital - Adults	Dr David Derry
Derriford Hospital - Paediatrics	Dr Alan Cade
Eastbourne District General Hospital - Paediatrics	Dr. Geeta Gopal
Genoa CF Centre - Adults	Dr Laura Minicucci
Genoa CF Centre - Paediatrics	Dr Laura Minicucci
Gloucestershire Royal Hospital - Paediatrics	Dr Mike Webb
Great Ormond Street Hospital - Paediatrics	Dr Colin Wallis
Hillingdon Hospital - Paediatrics	Dr Stephen Goldring
Hull Royal Infirmary - Paediatrics	Dr Ashwini Kotwal
James Cook University Hospital - Paediatrics	Dr Rajamanickam Jayaraj
James Paget Hospital - Paediatrics	Dr Caroline Kavanagh
Kettering General Hospital - Paediatrics	Dr Patti Rao
King's College Hospital - Paediatrics	Dr Gary Ruiz
King's Mill Hospital - Paediatrics	Dr Mike Yanney
Leicester Children's Hospital - Paediatrics	Dr Erol Gaillard
Leighton Hospital - Paediatrics	Dr Julie Ellison
Lincoln County Hospital - Paediatrics	Dr Amol Chingale
Macclesfield District General Hospital - Paediatrics	Dr Surendran Chandrasekaran
Musgrove Park Hospital - Paediatrics	Dr Alexandra Powell
New Cross Hospital - Paediatrics	Dr Rosie Rayner
Norfolk & Norwich University Hospital - Paediatrics	Dr Caroline Kavanagh
North Devon District Hospital - Paediatrics	Dr Dermot Dalton
Nottingham City Hospital - Adults	Dr Jane Dewar
Nottingham Children's Hospital - Paediatrics	Professor Alan Smyth
Oxford Children's Hospital - Paediatrics	Dr Jeremy Hull
Pilgrim Hospital - Paediatrics	Dr Margaret Crawford
Queen Alexandra Hospital - Paediatrics	Dr Hannah Buckley

**Participating Centre**	**Investigators**
Royal Alexandra Children's Hospital - Paediatrics	Dr Paul Seddon
Royal Berkshire Hospital - Paediatrics	Dr Claire Holt
Royal Brompton Hospital - Adults	Dr Nick Simmonds
Royal Brompton Hospital - Paediatrics	Professor Andrew Bush
Royal Cornwall Hospital - Paediatrics	Dr Anne Prendiville
Royal Derby Hospital - Paediatrics	Dr Nigel Ruggins
Royal Devon & Exeter Hospital - Adults	Dr Patrick Oades
Royal Devon & Exeter Hospital - Paediatrics	Dr Patrick Oades
Royal Hospital for Sick Children - Paediatrics	Professor Steve Cunningham
Royal Preston Hospital - Paediatrics	Dr Karnam Sugumar
Royal Shrewsbury Hospital - Paediatrics	Dr Martyn Rees
Royal United Hospital - Paediatrics	Dr Rebecca Winterson
Royal Victoria Infirmary - Adults	Dr Simon Doe
Royal Victoria Infirmary - Paediatrics	Dr Malcolm Brodie
Salisbury District Hospital - Paediatrics	Dr Robert Scott-Jupp
Sheffield Children's Hospital - Paediatrics	Dr Christopher Taylor
Southampton General Hospital - Adults	Dr Mary Carroll
Southampton General Hospital - Paediatrics	Dr Julian Legg
St James Hospital - Paediatrics	Dr Tim Lee
Torbay Hospital - Adults	Dr Lee Dobson
Torbay Hospital - Paediatrics	Dr Atanu Mukherjee
University Hospital North Staffordshire - Adults	Professor Warren Lenny
University Hospital North Staffordshire - Paediatrics	Professor Warren Lenny
University Hospital of Wales - Adults	Dr Ian Ketchell
University Hospital of Wales - Paediatrics	Dr Julian Forton
Walsgrave Hospital - Paediatrics	Dr Edward Simmonds
Warrington Hospital - Paediatrics	Dr Christopher Bedford
William Harvey Hospital - Paediatrics	Dr Ola Smith
Wythenshawe Hospital - Adults	Dr Naveen Rao
Wythenshawe Hospital - Paediatrics	Dr Naveen Rao
York Teaching Hospital - Paediatrics	Dr Murray Wheeler

Supplementary Appendix 1: Multiple imputation of missing resource utilisation data and utility values

Missing data was multiply imputed (m=25) through the use of chained equations.[1] The data was assumed to be missing at random. To account for non-normality in the distributions of costs and HRQoL, predictive mean matching was used. Missing cost data was imputed at the level of the category (inpatient care, outpatient visits, A&E visits, primary care etc.) and total costs was passively imputed as a sum of the individual cost domains for those with missing data. For HRQoL, missing data was imputed for the utility score for each of the time-points and QALYs were passively calculated. Predictors within both the cost and HRQoL multiple imputation models were patients age, sex, treatment arm, costs at baseline.

To account for statistical uncertainty and the correlation between costs and patient outcomes, the data was bootstrap sampled with replacement 2,000 times.[2] For each bootstrap sample, missing data was imputed[3] and incremental measures of cost and outcome were calculated using regression-analysis, controlling for baseline HRQoL, age (measured in days) and treatment arm. The regression analysis used ordinary least squares accounting for the multiply imputed data through the use of Rubin's rules.[4] All regression analysis including bootstrapping and multiple imputation was conducted within Stata v.14.

References

1. White IR, Royston P, Wood AM. Multiple imputation using chained equations: Issues and guidance for practice. Stat Med 2011;30(4):377-99.
2. Nixon RM, Wonderling D, Grieve RD. Non-parametric methods for cost-effectiveness analysis: the central limit theorem and the bootstrap compared. Health Econ 2010;19(3):316-33.
3. Schomaker M, Heumann C. Bootstrap inference when using multiple imputation. Stat Med 2018;37(14):2252-66.
4. Rubin DB, Schenker N. Multiple imputation in health-care databases: an overview and some applications. Stat Med 1991;10(4):585-98.

Supplementary Table 1: Secondary care unit costs used within the analysis

**Outpatients**	**Measurement**	**Unit cost**	**Source**	**Detail**
Non consultant-led, adult	Per visit	£75	NHS Reference costs 2016-2017[1]	Any non-paediatric outpatient, weighted average
Non consultant-led, child	Per visit	£149	NHS Reference costs 2016-2017[1]	Any paediatric outpatient, weighted average
Consultant-led, adult	Per visit	£136	NHS Reference costs 2016-2017[1]	Any non-paediatric outpatient, weighted average
Consultant-led, child	Per visit	£196	NHS Reference costs 2016-2017[1]	Any paediatric outpatient, weighted average
Dietician	Per visit	£37	PSSRU 2010 [2]	With qualification and including direct care costs, inflated to 2016-2017 using HCHS index
Physiotherapist	Per visit	£49	NHS Reference costs 2016-2017[1]]	
MDT, adult	Per visit	£1,541	NICE 2017 [3]	MDT-clinic, based on 250 adult patients per year, 6 visits per person per year
MDT, child	Per visit	£1,254	NICE 2017 [3]	MDT-clinic, based on 250 child patients per year, 6 visits per person per year
Radiology	Per visit	£135	NHS Reference costs 2016-2017[1]	Interventional Radiology
Pharmacy	Per visit		PSSRU 2010 [2]	With qualification and including direct care costs, inflated to 2016-2017 using HCHS index
A&E, discharged	Per visit	£128	NHS Reference costs 2016-2017[1]	Any A&E discharged, weighted average
A&E, admitted	Per visit	£221	NHS Reference costs 2016-2017 [1]	Any A&E admitted, weighted average
**Intervention**				
Ceftazidime	Per vial	£4·25 - £17·59	BNF 2017 [4]	
Tobramycin	Per vial	£19	BNF 2017 [4]	
Ciprofloxacin	Per tablet	£0·80 - £1·20	BNF 2017 [4]	
**Inpatient ward stays**				
General ward, adult	Per diem	£380	NHS Reference costs 2016-2017[1]	Excess bed days, weighted average for adults

General ward, child	Per diem	£595	NHS Reference costs 2016-2017[1]	Excess bed day, weighted average for paediatrics

References

1. National Schedule of Reference Costs 2016-2017. London, UK: Department of Health; 2-17.
2. Curtis L. Unit costs and social care. Personal Social Services Research Unit, University of Kent, Canterbury, 2010.
3. Cystic Fibrosis: diagnosis and management; Appendix K. London, UK: National Institute for Health and Care Excellence; 2017.
4. British National Formulary. Joint Formulary Committee; London, UK: BMJ Group and Pharmaceutical Press; 2017.

Supplementary Table 2: Unit costs in primary care or community care

	Measurement	Unit cost	Source	Detail
**Primary care**				
GP visit in surgery	Per visit	£38	PSSRU 2017[1]	With qualification and including direct care costs
Nurse visit in surgery	Per visit	£14	PSSRU 2017[1]	With qualifications and including direct care costs· Assuming 20 minutes consultation
Doctor in walk-in centre	Per visit	£44	NHS Reference costs 2016-2017[2]	Weighted average of non-admitted, Type 4 A&E visits
Nurse in walk-in centre	Per visit	£44	NHS Reference costs 2016-2017[2]	Weighted average of non-admitted, Type 4 A&E visits
Other	Per visit	£35		Average of above
**Home Visits**				
GP visit	Per home visit	£161	PSSRU 2017[1]	With qualification and including direct care costs, assuming 40 minutes with travel
District Nurse	Per home visit	£29	PSSRU 2010[3]	With qualification and including direct care costs, inflated to 2016-2017 using HCHS index
Health Visitor	Per home visit	£58	PSSRU 2010[3]	With qualification and including direct care costs, inflated to 2016-2017 using HCHS index
Nurse	Per home visit	£44	PSSRU 2010[3]	With qualification and including direct care costs, inflated to 2016-2017 using HCHS index
Physiotherapist	Per home visit	£61	PSSRU 2010[3]	With qualification and including direct care costs, inflated to 2016-2017 using HCHS index
Occupational therapy	Per home visit	£45	PSSRU 2017[1]	With qualification and including direct care costs
Other	Per home visit	£47		Average of all apart from GP visit
**Community based professional**				
Home care work	Per visit	£21	PSSRU 2017[1]	With qualification and including direct care costs, 1 hour
Nurse	Per visit	£44		Same as nurse home visit
Physiotherapist	Per visit	£49		Same as physiotherapist home visit
Social worker	Per visit	£57	PSSRU 2017[1]	With qualification and including direct care costs

References

1. Curtis L, Burns A. *Unit Costs of Health and Social Care 2017*. Personal Social Services Research Unit, University of Kent, Canterbury, 2017.
2. National Schedule of Reference Costs 2016-2017. London, UK: Department of Health; 2-17.
3. Curtis L. Unit costs and social care. Personal Social Services Research Unit, University of Kent, Canterbury, 2010.

Supplementary Table 3: Societal unit costs

	Measurement	Unit cost	Source	Detail
**Patient Aids**				
Anti-allergy pillow	Per item	£11	Wilko[1]	
Anti-allergy bed covers	Per item	£8	Wilko[2]	
Peak flow medicine	Per item	£5	BNF 2017[3]	
**Travel costs**				
Car	Per minute	£0·23	AA[4]	Based on the motoring costs per mile for domestic purposes
Bus	Per minute	£0·08	Department for Transport [5]	Local bus transport statistics reported· 3·5 miles per journey
Taxi	Per minute	£0·53	Local authority average	Assumption (based on average of Glasgow,[6] Liverpool,[7] Manchester[8] and Plymouth[9])
**Time lost from work**				
Carer / patient time	Per hour	£17	Office for National Statistics[10]	Work per minute using the mean hourly earnings reported by the Office of National Statistics
**Over the counter medicines**				
Various	Per medicine/pill	Various	BNF 2017[3]	

References

1. Anti allergy pillow. https://www.wilko.com/wilko-anti-allergy-medium-support-pillows-2pk/p/0478243 (accessed 31/07/2019).
2. Anti allergy bed covers. https://www.wilko.com/en-uk/wilko-king-size-anti-allergy-mattress-protector/p/0324832 (accessed 31/07/2019).
3. British National Formulary. Joint Formulary Committee; London, UK: BMJ Group and Pharmaceutical Press; 2017.
4. The NHS Staff Council. Pay Circular (AforC) 3/2014: amendment number 32. Annex 12: Motoring costs. https://www.nhsemployers.org/employershandbook/Annex-12-Motoring-costs.pdf (accessed 31/07/2019).
5. Department for Transport. Costs, fares and revenue (BUS04). https://www.gov.uk/government/statistical-data-sets/bus04-costs-fares-and-revenue (accessed 31/07/2019).
6. Glasgow taxi. https://www2.gov.scot/Topics/Government/Procurement/Selling/taxiglasgow (accessed 31/07/2019).
7. Liverpool taxi. https://liverpool.gov.uk/media/9294/tariff-sheet-2018.pdf (accessed 31/07/2019).
8. Manchester taxi. https://secure.manchester.gov.uk/downloads/download/3399/hackney_carriage_vehicle-fare_card_2011_2012 (accessed 31/07/2019).
9. Plymouth taxi. https://www.plymouth.gov.uk/parkingandtravel/publictransport/taxis) (accessed 31/07/2019).
10. ONS employment. https://www.ons.gov.uk/employmentandlabourmarket/peopleinwork/earningsandworkinghours/datasets/ashe1997to2015selectedestimates (accessed 31/07/2019).

Supplementary Table 4: Cystic Fibrosis Specialty HRG costs

	Measurement	Unit cost	Source
Band 1 adults 17 years and over	Per year	£3,362	NHS Reference costs 2016-2017 [39]
Band 1A adults 17 years and over	Per year	£5,380	NHS Reference costs 2016-2017
Band 1A children 16 years and under	Per year	£5,778	NHS Reference costs 2016-2017
Band 1 children 16 years and under	Per year	£5,685	NHS Reference costs 2016-2017
Band 2 adults 17 years and over	Per year	£6,498	NHS Reference costs 2016-2017
Band 2A adults 17 years and over	Per year	£8,922	NHS Reference costs 2016-2017
Band 2A children 16 years and under	Per year	£8,968	NHS Reference costs 2016-2017
Band 2 children 16 years and under	Per year	£7,492	NHS Reference costs 2016-2017
Band 3 adults 17 years and over	Per year	£15,337	NHS Reference costs 2016-2017
Band 3 children 16 years and under	Per year	£16,770	NHS Reference costs 2016-2017

References

1. National Schedule of Reference Costs 2016-2017. London, UK: Department of Health; 2-17.

Supplementary Table 5: Types of samples that confirmed participants had re-isolated *P. aeruginosa*

Sample type	IV antibiotic therapy (N=62)	Oral antibiotic therapy (N=70)
Bronchoscopy Lavage (BAL) & Cough swab	1 (1·4%)	0 (0%)
Cough plate	3 (4·3%)	2 (3·2%)
Cough swab	44 (62·9%)	37 (59·7%)
Cough swab & Sputum sample	0 (0%)	1 (1·6%)
Nose swab	1 (1·4%)	0 (0%)
Sputum sample (not induced)	18 (25·7%)	20 (32·3%)
Sputum sample (induced)	0 (0%)	1 (1·6%)
Throat swab	1 (1·4%)	1 (1·6%)
Other type	2 (2·9%)	0 (0%)

Supplementary Table 6: Samples that were resistant to any of the study drugs at re-isolation

Resistant to	IV antibiotic therapy (N=62)	Oral antibiotic therapy (N=70)
Ceftazidime	0 (0%)	1 (1·6%)
Tobramycin	1 (1·4%)	0 (0%)
Ciprofloxacin	1 (1·4%)	2 (3·2%)
Colistin	0 (0%)	0 (0%)

Supplementary Table 7: Analyses of primary outcome

Analysis	IV antibiotic therapy	Oral antibiotic therapy	Relative risk (95% CI)	p-value
Primary outcome: successful eradication of *P· aeruginosa* 3 months after treatment commencement and remaining infection free to 15 months				
Primary analysis	55/125 (44%)	68/130 (52·3%)	0·84 (0·65, 1·09)	0·18
Sensitivity analysis 1: All patients followed up past 3 months with no 15 month sample classified as success	66/136 (48·5%)	85/144 (56·9%)	0·85 (0·68, 1·07)	0·16
Sensitivity analysis 2: All patients followed up past 3 months with no 15 month sample classified as failure	55/136 (40·4%)	68/144 (47·2%)	0·86 (0·66, 1·12)	0·25
Sensitivity analysis 3: All patients followed up past 3 months with no 15 month sample classified as success/failure in accordance with the next sample taken after 15 month window	66/136 (48·5%)	81/144 (56·3%)	0·86 (0·69, 1·08)	0·20
Sensitivity analysis 4: As primary analysis but 3 and 15 month windows extended to -4 weeks/+10 weeks*	61/132 (46·2%)	73/139 (52·5%)	0·88 (0·69, 1·12)	0·30
Sensitivity analysis 5: As per primary analysis but 15-month window removed (any sample after 3-month window included)*	58/136 (42·7%)	70/144 (48·6%)	0·88 (0·68, 1·13)	0·32

Date are n/N (%), unless otherwise indicated

*Post hoc analysis

Supplementary Table 8: Analyses of secondary outcome ‘Time to reoccurrence of original P. aeruginosa infection’

Analysis	IV antibiotic therapy N=137	Oral antibiotic therapy N=148	Hazard ratio (95% CI)	p-value
Time to reoccurrence of original *P. aeruginosa* infection – unknown strains assumed to be the same as baseline				
Time from randomisation	74 (54·0%)	66 (44·6%)	1·37 (0·99, 1·91)	0·06
Time from treatment commencement	74 (54·0%)	66 (44·6%)	1·38 (0·99, 1·92)	0·06
Time to reoccurrence of original *P. aeruginosa* infection – unknown strains assumed to be different to baseline				
Time from randomisation	21 (15·3%)	14 (9·5%)	1·85 (0·94, 3·64)	0·08
Time from treatment commencement	21 (15·3%)	14 (9·5%)	1·85 (0·94, 3·64)	0·07

Date are n (%) for participants experiencing reoccurrence of the original *P. aeruginosa* infection, unless otherwise indicated

Supplementary Table 9: Analyses of secondary outcome ‘Re-infection with a different and distinct genotype of *P. aeruginosa*’

IV antibiotic therapy N=25	Oral antibiotic therapy N=17	Relative risk (95% CI)	p-value
6 (24·0%)	5 (29·4%)	0·82 (0·30, 2·25)	0·73

Date are n (%), unless otherwise indicated

Supplementary Table 10: Number of participants with an additional organism of interest during 15 months post randomisation

Organism	Intravenous antibiotic therapy N=137	Oral antibiotic therapy N=148
Staphylococcus aureus	51 (37·2%)	53 (35·8%)
Haemophilus influenza	36 (26·3%)	32 (21·6%)
Mycobacterium abscessus	0 (0%)	1 (0·7%)
Mycobacterium avium intracellulare complex	0 (0%)	0 (0%)
Other non-tuberculous mycobacteria	0 (0%)	0 (0%)
Stenotrophomonas maltophilia	6 (4·4%)	9 (6·1%)
Achromobacter xylosoxidans	1 (0·7%)	3 (20·0%)

Data are n (%), unless otherwise indicated

Supplementary Table 11: Estimates from random effects models for CFQ-R

Domain	N	Mean treatment difference at 15 months (95% CI)	p-value
Self-report questionnaire			
Physical functioning	106	-3·63 (-10·41, 3·16)	0·29
Role/school functioning*	27	7·66 (-6·21, 21·52)	0·27
Vitality*	27	5·4 (-6·88, 17·69)	0·37
Emotional functioning	106	-1·59 (-7·39, 4·22)	0·59
Social functioning	106	2·11 (-4·03, 8·25)	0·50
Body image	105	-4·01 (-11·78, 3·77)	0·31
Eating problems	106	-0·39 (-6·78, 6)	0·90
Treatment burden	105	2·86 (-6·19, 11·92)	0·53
Health perceptions*	27	5·06 (-13·72, 23·84)	0·58
Weight*	27	1·4 (-19·02, 21·83)	0·89
Respiratory symptoms	106	2·82 (-3·44, 9·08)	0·37
Digestive symptoms	106	-0·01 (-9·95, 9·93)	0·99
Parent/carer questionnaire			
Physical functioning	73	-5·17 (-13·55, 3·22)	0·22
Role/school functioning	72	-1·47 (-10·22, 7·28)	0·74
Vitality	72	-0·44 (-9·36, 8·48)	0·92
Emotional functioning	72	3·32 (-3·56, 10·2)	0·34
Body image	72	-0·56 (12·03, 10·9)	0·92
Eating problems	72	6·15 (-2·78, 15·08)	0·17
Treatment burden	73	-0·28 (-11·25, 10·69)	0·96
Health perceptions	72	-7·25 (-15·09, 0·6)	0·07
Weight	73	-1·19 (-17·63, 15·26)	0·89
Respiratory symptoms	73	-3·33 (-11·71, 9·93)	0·43
Digestive symptoms	72	3·54 (-4·9, 11·98)	0·41

*: Only asked in participants aged 14 and over

Supplementary Table 12: Median number of days' absence during the 15 months following randomisation for carer and participant (absenteeism from education or work)

	Treatment	N	Median	IQR	Min, Max	p-value
Carer	IV antibiotic therapy	131	0	(0,1)	0,24	0·62
	Oral antibiotic therapy	139	0	(0,1)	0,98	
Participant	IV antibiotic therapy	131	0	(0,6·2)	0,56	0.26
	Oral antibiotic therapy	140	1	(0,10)	0,113	

Supplementary Table 13: All non-serious adverse events

Preferred Term	IV Antibiotic Therapy N=126		Oral Antibiotic Therapy N=146		Total N=272	
	Events	Patients	Events	Patients	Events	Patients
Any non-serious adverse event[ab]	126	60 (47·6%)	136	72(49·3%)	262	132 (48·5%)
Cough	26	22 (17·5%)	28	23 (15·8%)	54	45 (16·5%)
Upper respiratory tract infection	15	11 (8·7%)	3	2 (1·4%)	18	13 (4·8%)
Productive cough	5	5 (4%)	8	8 (5·5%)	13	13 (4·8%)
Diarrhoea	6	5 (4%)	3	3 (2·1%)	9	8 (2·9%)
Pyrexia	2	2 (1·6%)	7	7 (4·8%)	9	9 (3·3%)
Wheezing	3	3 (2·4%)	6	6 (4·1%)	9	9 (3·3%)
Infective pulmonary exacerbation of cystic fibrosis[b]	4	3 (2·4%)	3	3 (2·1%)	7	6 (2·2%)

Preferred Term	IV Antibiotic Therapy N=126		Oral Antibiotic Therapy N=146		Total N=272	
	Events	Patients	Events	Patients	Events	Patients
Candida infection	1	1 (0·8%)	5	5 (3·4%)	6	6 (2·2%)
Distal intestinal obstruction syndrome	2	2 (1·6%)	3	3 (2·1%)	5	5 (1·8%)
Varicella	3	3 (2·4%)	1	1 (0·7%)	4	4 (1·5%)
Abdominal pain	1	1 (0·8%)	2	2 (1·4%)	3	3 (1·1%)
Arthralgia	0	0 (0%)	3	3 (2·1%)	3	3 (1·1%)
Epistaxis	1	1 (0·8%)	2	2 (1·4%)	3	3 (1·1%)
Fall	1	1 (0·8%)	2	2 (1·4%)	3	3 (1·1%)
Haemophilus influenza respiratory infection[c]	3	3 (2·4%)	0	0 (0%)	3	3 (1·1%)
Headache	3	2 (1·6%)	0	0 (0%)	3	2 (0·7%)
Pulmonary function decreased[c]	1	1 (0·8%)	2	2 (1·4%)	3	3 (1·1%)
Rash	2	2 (1·6%)	1	1 (0·7%)	3	3 (1·1%)
Respiratory tract infection	1	1 (0·8%)	2	2 (1·4%)	3	3 (1·1%)
Vomiting	3	3 (2·4%)	0	0 (0%)	3	3 (1·1%)
Abdominal pain upper	1	1 (0·8%)	1	1 (0·7%)	2	2 (0·7%)
Adverse drug reaction	1	1 (0·8%)	1	1 (0·7%)	2	2 (0·7%)
Bronchospasm	1	1 (0·8%)	1	1 (0·7%)	2	2 (0·7%)
Chest pain	1	1 (0·8%)	1	1 (0·7%)	2	2 (0·7%)
Constipation	1	1 (0·8%)	1	1 (0·7%)	2	2 (0·7%)
Dermatitis diaper	1	1 (0·8%)	1	1 (0·7%)	2	2 (0·7%)
Device occlusion	2	2 (1·6%)	0	0 (0%)	2	2 (0·7%)
Dizziness	1	1 (0·8%)	1	1 (0·7%)	2	2 (0·7%)
Ear pain	0	0 (0%)	2	2 (1·4%)	2	2 (0·7%)
Nausea	1	1 (0·8%)	1	1 (0·7%)	2	2 (0·7%)
Pain	1	1 (0·8%)	1	1 (0·7%)	2	2 (0·7%)
Seasonal allergy	1	1 (0·8%)	1	1 (0·7%)	2	2 (0·7%)
Viral infection	1	1 (0·8%)	1	1 (0·7%)	2	2 (0·7%)
Vulvovaginal candidiasis	2	2 (1·6%)	0	0 (0%)	2	2 (0·7%)
Administration site bruise	1	1 (0·8%)	0	0 (0%)	1	1 (0·4%)
Administration site pain	1	1 (0·8%)	0	0 (0%)	1	1 (0·4%)
Alanine aminotransferase increased	1	1 (0·8%)	0	0 (0%)	1	1 (0·4%)
Allergic bronchopulmonary aspergillosis (ABPA)[c]	0	0 (0%)	1	1 (0·7%)	1	1 (0·4%)
Back pain	1	1 (0·8%)	0	0 (0%)	1	1 (0·4%)
Blood glucose increased	1	1 (0·8%)	0	0 (0%)	1	1 (0·4%)
Catheter site related reaction	1	1 (0·8%)	0	0 (0%)	1	1 (0·4%)
Chest infection[c]	0	0 (0%)	1	1 (0·7%)	1	1 (0·4%)
Chest X-ray abnormal	0	0 (0%)	1	1 (0·7%)	1	1 (0·4%)
Conjunctivitis	1	1 (0·8%)	0	0 (0%)	1	1 (0·4%)
Decreased appetite	0	0 (0%)	1	1 (0·7%)	1	1 (0·4%)
Dry skin	1	1 (0·8%)	0	0 (0%)	1	1 (0·4%)
Dysuria	0	0 (0%)	1	1 (0·7%)	1	1 (0·4%)
Ear discomfort	1	1 (0·8%)	0	0 (0%)	1	1 (0·4%)
Eczema	1	1 (0·8%)	0	0 (0%)	1	1 (0·4%)
Eczema infected	0	0 (0%)	1	1 (0·7%)	1	1 (0·4%)
Enterobacter cloacae respiratory infection[c]	1	1 (0·8%)	0	0 (0%)	1	1 (0·4%)
Enuresis	0	0 (0%)	1	1 (0·7%)	1	1 (0·4%)

Preferred Term	IV Antibiotic Therapy N=126		Oral Antibiotic Therapy N=146		Total N=272	
	Events	Patients	Events	Patients	Events	Patients
Eye infection	1	1 (0·8%)	0	0 (0%)	1	1 (0·4%)
Febrile convulsion	0	0 (0%)	1	1 (0·7%)	1	1 (0·4%)
Flank pain	0	0 (0%)	1	1 (0·7%)	1	1 (0·4%)
Gastroenteritis	0	0 (0%)	1	1 (0·7%)	1	1 (0·4%)
Haematemesis	0	0 (0%)	1	1 (0·7%)	1	1 (0·4%)
Haemophilus parainfluenza respiratory infection[c]	1	1 (0·8%)	0	0 (0%)	1	1 (0·4%)
Haemoptysis	1	1 (0·8%)	0	0 (0%)	1	1 (0·4%)
Hand, foot and mouth disease (recorded as foot and mouth disease)[cd]	0	0 (0%)	1	1 (0·7%)	1	1 (0·4%)
Hand, foot and mouth disease[c]	0	0 (0%)	1	1 (0·7%)	1	1 (0·4%)
Infectious mononucleosis	0	0 (0%)	1	1 (0·7%)	1	1 (0·4%)
Influenza like illness	0	0 (0%)	1	1 (0·7%)	1	1 (0·4%)
Klebsiella pneumoniae respiratory infection[c]	0	0 (0%)	1	1 (0·7%)	1	1 (0·4%)
Lethargy	0	0 (0%)	1	1 (0·7%)	1	1 (0·4%)
Limb discomfort	1	1 (0·8%)	0	0 (0%)	1	1 (0·4%)
Malaise	0	0 (0%)	1	1 (0·7%)	1	1 (0·4%)
Malignant melanoma	0	0 (0%)	1	1 (0·7%)	1	1 (0·4%)
Migraine	0	0 (0%)	1	1 (0·7%)	1	1 (0·4%)
Musculoskeletal chest pain	0	0 (0%)	1	1 (0·7%)	1	1 (0·4%)
Musculoskeletal stiffness	0	0 (0%)	1	1 (0·7%)	1	1 (0·4%)
Myalgia	0	0 (0%)	1	1 (0·7%)	1	1 (0·4%)
Mycobacterium avium complex infection[e]	0	0 (0%)	1	1 (0·7%)	1	1 (0·4%)
Nasal congestion	1	1 (0·8%)	0	0 (0%)	1	1 (0·4%)
Nasal swelling[c]	0	0 (0%)	1	1 (0·7%)	1	1 (0·4%)
Nasal vestibulitis	1	1 (0·8%)	0	0 (0%)	1	1 (0·4%)
Nasopharyngitis	1	1 (0·8%)	0	0 (0%)	1	1 (0·4%)
Onychoclasis	1	1 (0·8%)	0	0 (0%)	1	1 (0·4%)
Oral candidiasis	0	0 (0%)	1	1 (0·7%)	1	1 (0·4%)
Otitis media	1	1 (0·8%)	0	0 (0%)	1	1 (0·4%)
Pain in extremity	0	0 (0%)	1	1 (0·7%)	1	1 (0·4%)
Pancreatitis	1	1 (0·8%)	0	0 (0%)	1	1 (0·4%)
Paraesthesia oral	0	0 (0%)	1	1 (0·7%)	1	1 (0·4%)
Petechiae	0	0 (0%)	1	1 (0·7%)	1	1 (0·4%)
Pharyngeal oedema	0	0 (0%)	1	1 (0·7%)	1	1 (0·4%)
Photosensitivity reaction	0	0 (0%)	1	1 (0·7%)	1	1 (0·4%)
Pneumonia	1	1 (0·8%)	0	0 (0%)	1	1 (0·4%)
Polyuria	1	1 (0·8%)	0	0 (0%)	1	1 (0·4%)
Rectal haemorrhage	0	0 (0%)	1	1 (0·7%)	1	1 (0·4%)
Sinusitis	1	1 (0·8%)	0	0 (0%)	1	1 (0·4%)
Skin discolouration	0	0 (0%)	1	1 (0·7%)	1	1 (0·4%)
Skull fracture	0	0 (0%)	1	1 (0·7%)	1	1 (0·4%)
Sputum increased	1	1 (0·8%)	0	0 (0%)	1	1 (0·4%)
Stenotrophomonas maltophilia respiratory infection[c]	0	0 (0%)	1	1 (0·7%)	1	1 (0·4%)
Streptococcus pyogenes respiratory infection[c]	0	0 (0%)	1	1 (0·7%)	1	1 (0·4%)
Sunburn	0	0 (0%)	1	1 (0·7%)	1	1 (0·4%)
Tendonitis	0	0 (0%)	1	1 (0·7%)	1	1 (0·4%)
Tongue discolouration	1	1 (0·8%)	0	0 (0%)	1	1 (0·4%)
Urinary tract infection	1	1 (0·8%)	0	0 (0%)	1	1 (0·4%)

Preferred Term	IV Antibiotic Therapy N=126		Oral Antibiotic Therapy N=146		Total N=272	
	Events	Patients	Events	Patients	Events	Patients
Urticaria	0	0 (0%)	1	1 (0·7%)	1	1 (0·4%)
Vitamin A deficiency	0	0 (0%)	1	1 (0·7%)	1	1 (0·4%)
Vitamin D deficiency	0	0 (0%)	1	1 (0·7%)	1	1 (0·4%)
Vitamin E deficiency	0	0 (0%)	1	1 (0·7%)	1	1 (0·4%)
Wrist fracture	1	1 (0·8%)	0	0 (0%)	1	1 (0·4%)

Data are n or n (%).

[a] 9 additional adverse events were reported by 9 participants in the IV antibiotic therapy group and 5 adverse events from 5 participants in the oral antibiotic therapy group but have not been included here as they were the event of interest for the primary outcome so should not have been reported as an adverse event.

[b] 3 additional adverse events were reported by 3 participants in the oral antibiotic therapy group but have not been included here as they contributed to the analysis of the outcome ‘Number of pulmonary exacerbations’.

[c] Wording not consistent with ‘preferred term’ recorded on database, amended based on additional data available.

[d] Not confirmed in medical notes

[e] Not reported on the microbiology CRF for the sample taken on the date of onset so not included in Supplementary Table 10.

Supplementary Table 14: Mean unadjusted costs based on 15-month time horizon and outcomes between trial arms based on multiply imputed data (imputations=25)

	**Intravenous antibiotic therapy (IVT), mean value, (standard error)<br><br>(n=125)**	**Oral antibiotic therapy (OT), mean value, (standard error)<br><br>(n=130)**	**Incremental (OT - IVT)***
**NHS resource use**			
A&E	£24·15 (7.69)	£23·79 (7.55)	
Inpatients	£1027·0 (338.0)	£1682·4 (390.9)	
Outpatients	£1221·5 (121.0)	£1414·0 (120.5)	
Primary care	£28·42 (10.92)	£25·13 (6.48)	
Home visits	£185·1 (35.6)	£172·0 (30.54)	
Prescribed medicines	£124·3 (63.76)	£248·2 (86.67)	
Total costs (not including intervention)	£2610·5 (401.3)	£3565·4 (450.5)	954·9 (95% CI:-132·1, 2041·9)
Intervention cost	£7284·4 (242.0)	£264·3 (124.1)	-7020·2 (95% CI:-7548·5, -6491·8)
Total NHS costs (including intervention)	£9895·0 (472.2)	£3829·7 (457.5)	-6065·3 (95% CI:-7287·1, -4843·4)
**Wider resource use**			
Patient/carer lost work	£7·67 (3.13)	£1·88 (0.73)	
Aids & Appliances	£8·77 (3.73)	£11·13 (2.86)	
Over counter medicine	£1·01 (0.301)	£1·04 (0.212)	
Travel costs	£86·05 (22.17)	£90·49 (18.01)	
Total societal costs (plus intervention)	£9998·5 (476.1)	£3934·3 (462.0)	-6064·2 (95% CI:-7296·2, -4832·2)
**Outcomes**			
Primary outcome (% successful eradication)	0·44 (0.05)	0·52 (0.04)	0·083 (95% CI:-0·040, 0·21)
Health related quality of life (HRQoL)			
EQ5D baseline	0·820 (0.02)	0·873 (0.02)	
EQ5D 3 months	0·854 (0.02)	0·893 (0.02)	
EQ5D 15 months	0·829 (0.03)	0·893 (0.02)	
EQ5D 24 months	0·895 (0.02)	0·923 (0.02)	
Quality-adjusted life-years (over 15-months)	1·050 (0.02)	1·114 (0.02)	0·063 (95% CI:0·0074, 0·12)
Quality-adjusted life-years (over 24-months)	1·697 (0.03)	1·795 (0.03)	0·098 (95% CI:0·013, 0·18)

*Unadjusted for baseline characteristics, age and baseline HRQoL.

Supplementary Table 15: NHS and PSS resource use and missingness data (over 15-months)

	IV Antibiotic Therapy N=125	Oral Antibiotic Therapy N=130
**Secondary Care**		
A&E attendance, admitted		
0 visits	54 (91.5%)	61 (88.4%)
1 visit	3 (5.1%)	8 (11.6%)
2 visits	2 (3.4%)	0 (0.0%)
3+ visits	0 (0.0%)	0 (0.0%)
All Missing	66	61
A&E attendance, discharged		
0 visits	57 (96.6%)	66 (95.7%)
1 visit	1 (1.7%)	2 (2.9%)
2 visits	1 (1.7%)	1 (1.4%)
3+ visits	0 (0.0%)	0 (0.0%)
All Missing	66	61
Inpatient stay		
0 visits	66 (80.5%)	65 (72.2%)
1 visit	11 (13.4%)	19 (21.1%)
2 visits	4 (4.9%)	3 (3.3%)
3+ visits	1 (1.2%)	3 (3.3%)
All Missing	43	40
Outpatient (visits)		
0 visits	0 (0.0%)	0 (0.0%)
1 visit	28 (59.6%)	29 (60.4%)
2 visits	11 (23.4%)	10 (20.8%)
3+ visits	8 (17.0%)	9 (18.8%)
All Missing	78	82
**Primary Care**		
GP at surgery		
0 visits	70 (82.4%)	78 (81.3%)
1 visit	11 (12.9%)	10 (10.4%)
2 visits	2 (2.4%)	5 (5.2%)
3+ visits	2 (2.4%)	3 (3.1%)
All Missing	40	34
Doctor at walk-in centre		
0 visits	85 (100.0%)	92 (95.8%)
1 visit	0 (0.0%)	3 (3.1%)
2 visits	0 (0.0%)	1 (1.0%)
3+ visits	0 (0.0%)	0 (0.0%)
All Missing	40	34
Nurse at surgery		
0 visits	77 (90.6%)	86 (89.6%)
1 visit	8 (9.4%)	9 (9.4%)
2 visits	0 (0.0%)	0 (0.0%)
3+ visits	0 (0.0%)	1 (1.0%)
All Missing	40	34

	IV Antibiotic Therapy N=125	Oral Antibiotic Therapy N=130
Nurse at walk-in centre		
0 visits	85 (100.0%)	96 (100.0%)
1 visit	0 (0.0%)	1 (1.0%)
2 visits	0 (0.0%)	1 (1.0%)
3+ visits	0 (0.0%)	1 (1.0%)
All Missing	40	34
Other		
0 visits	76 (89.4%)	89 (92.7%)
1 visit	6 (7.1%)	3 (3.1%)
2 visits	1 (1.2%)	2 (2.1%)
3+ visits	2 (2.4%)	2 (2.1%)
All Missing	40	34
Number of prescriptions	2.02 (2.79)	2.18 (3.02)
Missing	41	39
**Home visits**		
GP		
0 visits	36 (61.0%)	51 (69.9%)
1 visit	23 (39.0%)	22 (30.1%)
2 visits	0 (0.0%)	0 (0.0%)
3+ visits	0 (0.0%)	0 (0.0%)
All Missing	66	57
District Nurse		
0 visits	36 (61.0%)	51 (69.9%)
1 visit	21 (35.6%)	21 (28.8%)
2 visits	1 (1.7%)	1 (1.4%)
3+ visits	1 (1.7%)	0 (0.0%)
All Missing	66	57
Health Visitor		
0 visits	36 (61.0%)	51 (69.9%)
1 visit	21 (35.6%)	20 (27.4%)
2 visits	1 (1.7%)	0 (0.0%)
3+ visits	1 (1.7%)	2 (2.7%)
All Missing	66	57
Nurse		
0 visits	36 (61.0%)	50 (68.5%)
1 visit	15 (25.4%)	18 (24.7%)
2 visits	6 (10.2%)	1 (1.4%)
3+ visits	2 (3.4%)	4 (5.5%)
All Missing	66	57
Physiotherapy		
0 visits	36 (61.0%)	51 (69.9%)
1 visit	19 (32.2%)	20 (27.4%)
2 visits	2 (3.4%)	1 (1.4%)
3+ visits	2 (3.4%)	1 (1.4%)
All Missing	66	57

	IV Antibiotic Therapy N=125	Oral Antibiotic Therapy N=130
Occupational therapy		
0 visits	36 (61.0%)	51 (69.9%)
1 visit	23 (39.0%)	22 (30.1%)
2 visits	0 (0.0%)	0 (0.0%)
3+ visits	0 (0.0%)	0 (0.0%)
All Missing	66	57
Other		
0 visits	36 (61.0%)	51 (69.9%)
1 visit	23 (39.0%)	22 (30.1%)
2 visits	0 (0.0%)	0 (0.0%)
3+ visits	0 (0.0%)	0 (0.0%)
All Missing	66	57
Home care worker		
0 visits	55 (96.5%)	63 (90.0%)
1 visit	0 (0.0%)	0 (0.0%)
2 visits	2 (3.5%)	7 (10.0%)
3+ visits	0 (0.0%)	0 (0.0%)
All Missing	68	60
**Community Based Professionals**		
Social worker		
0 visits	55 (96.5%)	63 (90.0%)
1 visit	0 (0.0%)	0 (0.0%)
2 visits	2 (3.5%)	7 (10.0%)
3+ visits	0 (0.0%)	0 (0.0%)
All Missing	68	60
Nurse		
0 visits	54 (94.7%)	67 (95.7%)
1 visit	1 (1.8%)	2 (2.9%)
2 visits	1 (1.8%)	1 (1.4%)
3+ visits	1 (1.8%)	0 (0.0%)
All Missing	68	60
Physiotherapist		
0 visits	57 (100.0%)	67 (95.7%)
1 visit	0 (0.0%)	2 (2.9%)
2 visits	0 (0.0%)	0 (0.0%)
3+ visits	0 (0.0%)	1 (1.4%)
All Missing	68	60

Supplementary Table 16: Societal resource utilisation and missingness

	IV Antibiotic Therapy N=125	Oral Antibiotic Therapy N=130
Patient aids		
0 aids	43 (74.1%)	54 (78.3%)
1 aid	0 (0.0%)	0 (0.0%)
2 aids	0 (0.0%)	1 (1.4%)
3+ aids	15 (25.9%)	15 (21.7%)
All Missing	67	61
Transport car		
No trips	1 (0.8%)	5 (4.0%)
0-5 trips	51 (41.5%)	49 (39.2%)
6-10 trips	7 (5.7%)	13 (10.4%)
More than 10 trips	64 (52.0%)	58 (46.4%)
All Missing	2	5
Transport bus		
No trips	29 (23.2%)	39 (30.0%)
0-5 trips	2 (1.6%)	3 (2.3%)
6-10 trips	0 (0.0%)	1 (0.8%)
More than 10 trips	94 (75.2%)	87 (66.9%)
All Missing	0	1
Transport taxi		
No trips	26 (20.8%)	37 (28.5%)
0-5 trips	9 (7.2%)	10 (7.7%)
6-10 trips	1 (0.8%)	0 (0.0%)
More than 10 trips	89 (71.2%)	83 (63.8%)
All Missing	0	1
Transport other		
No trips	21 (16.8%)	30 (23.3%)
0-5 trips	20 (16.0%)	14 (10.9%)
6-10 trips	0 (0.0%)	2 (1.6%)
More than 10 trips	84 (67.2%)	83 (64.3%)
All Missing	0	1
Over counter medicines		
0 purchases	110 (88.0%)	102 (78.5%)
1 purchase	7 (5.6%)	19 (14.6%)
2 purchase	6 (4.8%)	8 (6.2%)
3 purchase	2 (1.6%)	1 (0.8%)
Reported as losing time from work (patient)		
0	116 (99.1%)	124 (100.0%)
1	1 (0.9%)	0 (0.0%)
Reported as losing time from work (carer)		
0	106 (90.6%)	115 (92.7%)
1	11 (9.4%)	9 (7.3%)
All Missing	8	16

Supplementary Table 17: EQ-5D-3L reporting by time-point

EQ-5D-3L domain & level	Baseline		3-months		15-months		24-months	
	IV Antibiotic Therapy	Oral Antibiotic Therapy	IV Antibiotic Therapy	Oral Antibiotic Therapy	IV Antibiotic Therapy	Oral Antibiotic Therapy	IV Antibiotic Therapy	Oral Antibiotic Therapy
**Mobility**	N=96	N=109	N=89	N=101	N=73	N=95	N=59	N=68
**No problems**	90 (93.8%)	104 (95.4%)	83 (93.3%)	96 (94.1%)	60 (82.2%)	89 (93.7%)	53 (89.8%)	64 (94.1%)
**Some problems**	6 (6.3%)	5 (4.6%)	6 (6.7%)	6 (5.9%)	13 (17.8%)	6 (6.3%)	6 (10.2%)	4 (5.9%)
**Extreme problems**	0 (0.0%)	0(0.0%)	0 (0.0%)	0 (0.0%)	0 (0.0%)	0 (0.0%)	0 (0.0%)	0 (0.0%)
**Self-care**	N=91	N=103	N=86	N=99	N=67	N=90	N=57	N=67
**No problems**	67 (73.6%)	87 (84.5%)	67 (77.9%)	84 (84.8%)	55 (75.3%)	78 (86.7%)	48 (84.2%)	61 (89.7%)
**Some problems**	13 (14.3%)	5 (4.9%)	10 (11.6%)	6 (6.1%)	12 (16.4%)	8 (8.9%)	7 (12.3%)	4 (5.9%)
**Extreme problems**	11 (12.1%)	11 (10.7%)	9 (10.5%)	9 (9.1%)	6 (8.2%)	4 (4.4%)	2 (3.5%)	3 (4.4%)
**Usual Activities**	N=96	N=108	N=89	N=99	N=70	N=94	N=58	N=66
**No problems**	81 (84.4%)	101 (93.5%)	81 (91.0%)	89 (89.9%)	57 (80.3%)	84 (89.4%)	53 (91.4%)	62 (93.9%)
**Some problems**	13 (13.5%)	7 (6.5%)	7 (7.9%)	10 (10.1%)	13 (18.3%)	10 (10.6%)	4 (6.9%)	4 (6.1%)
**Extreme problems**	2 (2.1%)	0 (0.0%)	1 (1.1%)	0 (0.0%)	1 (1.4%)	0 (0.0%)	1 (1.7%)	0 (0.0%)
**Pain & Discomfort**	N=95	N=109	N=89	N=100	N=71	N=94	N=58	N=68
**No problems**	76 (80.0%)	89 (81.7%)	73 (82.0%)	82 (82.0%)	54 (74.0%)	77 (81.9%)	44 (75.9%)	59 (86.8%)
**Some problems**	19 (20.0%)	19 (17.4%)	16 (18.0%)	18 (18.0%)	17 (23.3%)	17 (18.1%)	14 (24.1%)	9 (13.2%)
**Extreme problems**	0.0%	1 (0.9%)	0 (0.0%)	0 (0.0%)	2 (2.7%)	0 (0.0%)	0 (0.0%)	0 (0.0%)
**Anxiety & Depression**	N=95	N=108	N=88	N=99	N=71	N=94	N=59	N=66
**No problems**	82 (86.3%)	96 (88.9%)	75 (85.2%)	88 (88.9%)	58 (80.6%)	82 (87.2%)	50 (84.7%)	63 (95.5%)
**Some problems**	12 (12.6%)	12 (11.1%)	11 (12.5%)	11 (11.1%)	13 (18.1%)	11 (11.7%)	9 (15.3%)	2 (3.0%)
**Extreme problems**	1 (1.1%)	0 (0.0%)	2 (2.3%)	0 (0.0%)	1 (1.4%)	1 (1.1%)	0 (0.0%)	1 (1.5%)

Supplementary Table 18: Incremental costs, outcomes, ICERs and incremental net benefit between trial arms for the sensitivity analysis

	**Incremental Cost^ £**	**Incremental Outcome^**	**ICER**	**Incremental net monetary benefit* £**
Primary analysis - % successful eradication, NHS and PSS perspective costs, 15-month horizon, covariate adjusted, Cystic Fibrosis HRG used	-653·08 (95% CI:-1197·8, -79·8)	0·091 (95% CI:-0·034, 0·22)	Oral dominates	N/A**
Primary analysis - % successful eradication, societal perspective costs, 15-month horizon, adjusted	-5937·0 (95% CI: -7120·8, -4659·1)	0·091 (95% CI:-0·034, 0·22)	Oral dominates	N/A**
Secondary analysis - 24-month horizon QALYs, NHS and PSS perspective costs, covariate adjusted	-5938·5 (95% CI:-7107·4, -4666·3)	0·058 (95% CI:-0·004, 0·140)	Oral dominates	7229·8 (95% CI: 5411·8, 8553·1)
Secondary analysis - 15-month horizon QALYs, NHS and PSS perspective costs, covariate, Cystic Fibrosis HRG used	-653·08 (95% CI:-1197·8, -79·8)	0·035 (95% CI:-0·007, 0·088)	Oral dominates	653·1 (95% CI: 79·8, 1197·8)
Secondary analysis - 15 months QALYs, societal perspective costs, 15-month horizon, covariate	-5937·0 (95% CI: -7052·6, -4713·2)	0·035 (95% CI:-0·007, 0·088)	Oral dominates	6755·7 (95% CI: 4977·4 to 8409·3)

^Adjusting for baseline EQ5D and age (in days) in linear regression
*At a cost-effectiveness threshold of £20,000 per QALY
**There is no societally accepted willingness-to-pay threshold associated with the primary outcome measure and so this is not reported.

Supplementary Figure 1a and Figure 1b: Incremental cost-effectiveness plane (top) and cost-effectiveness acceptability curve (bottom). Based on: % successful eradication, NHS and PSS perspective costs, covariate adjusted*

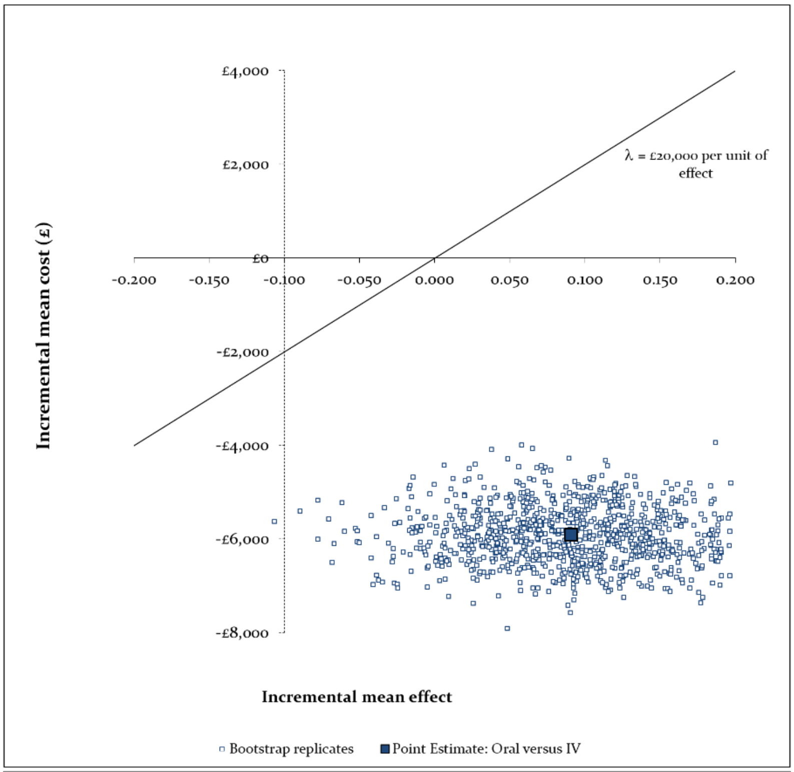

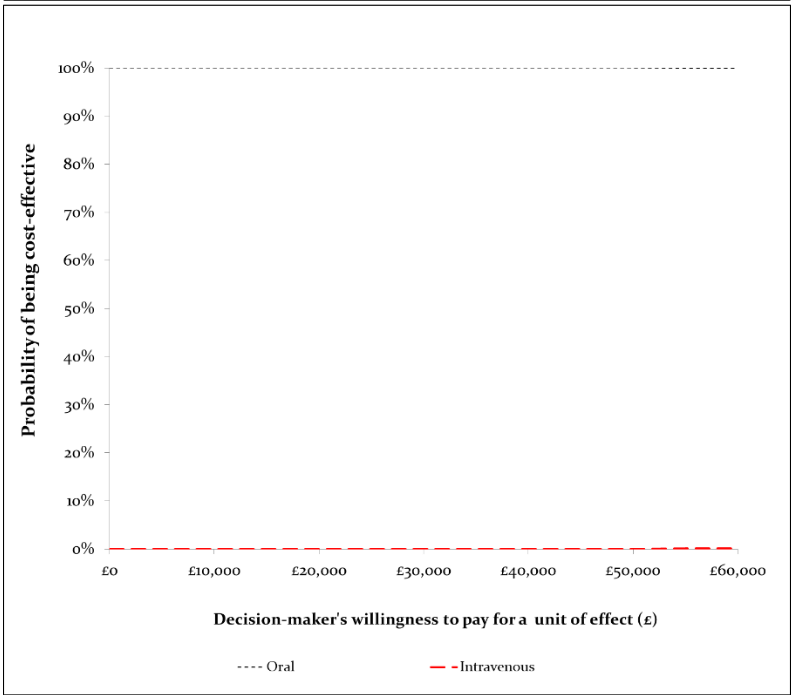

*Top figure: Blue squares represent bootstrap samples (n=2000) from the data for incremental cost and incremental QALYs for oral versus intravenous· The large square represents mean point estimates for incremental costs and QALYs· Squares falling below the threshold line suggest that oral is cost-effective versus intravenous for the given threshold.

Supplementary Figure 2a and 2b: Incremental cost-effectiveness plane (top) and cost-effectiveness acceptability curve (bottom). Based on: 15-month horizon QALYs, NHS and PSS perspective costs, covariate-adjusted*

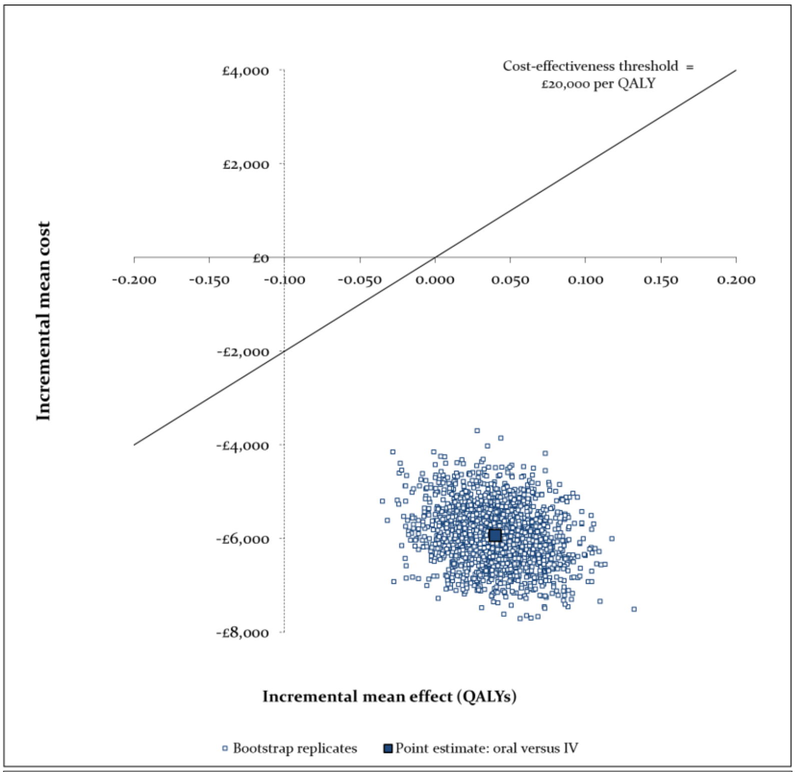

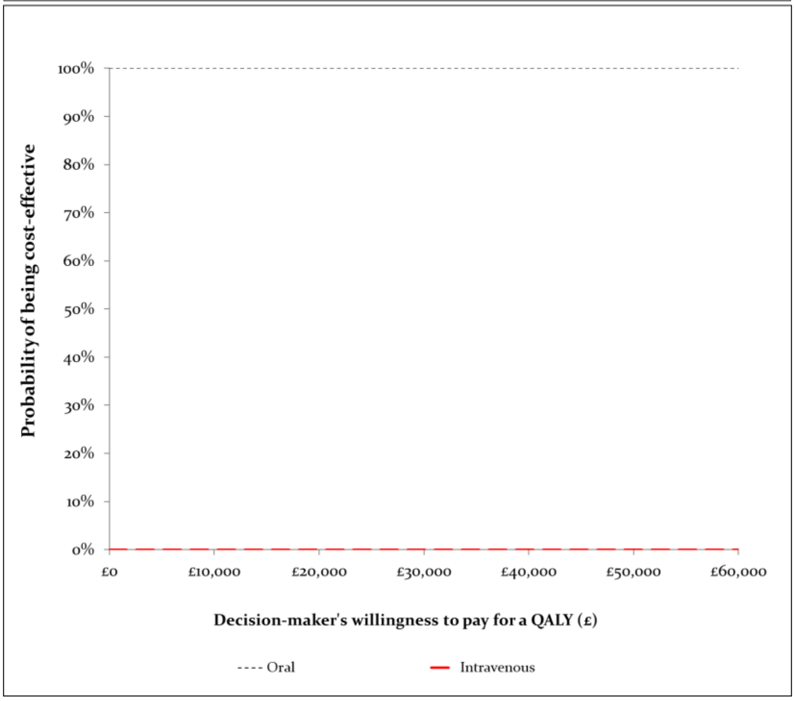

*Top figure: Blue squares represent bootstrap samples (n=2000) from the data for incremental cost and incremental QALYs for oral versus intravenous· The large square represents mean point estimates for incremental costs and QALYs· Squares falling below the threshold line suggest that oral is cost-effective versus intravenous for the given threshold. The threshold line is plotted for £20,000 per QALY as typically used by the National Institute for Health and Care Excellence (NICE).
